# Simulation Studies as Designed Experiments: The Comparison of Penalized Regression Models in the “Large *p,* Small *n*” Setting

**DOI:** 10.1371/journal.pone.0107957

**Published:** 2014-10-07

**Authors:** Elias Chaibub Neto, J. Christopher Bare, Adam A. Margolin

**Affiliations:** Sage Bionetworks, Seattle, Washington, United States of America; Thomas J. Watson Research Center, United States of America

## Abstract

New algorithms are continuously proposed in computational biology. Performance evaluation of novel methods is important in practice. Nonetheless, the field experiences a lack of rigorous methodology aimed to systematically and objectively evaluate competing approaches. Simulation studies are frequently used to show that a particular method outperforms another. Often times, however, simulation studies are not well designed, and it is hard to characterize the particular conditions under which different methods perform better. In this paper we propose the adoption of well established techniques in the design of computer and physical experiments for developing effective simulation studies. By following best practices in planning of experiments we are better able to understand the strengths and weaknesses of competing algorithms leading to more informed decisions about which method to use for a particular task. We illustrate the application of our proposed simulation framework with a detailed comparison of the ridge-regression, lasso and elastic-net algorithms in a large scale study investigating the effects on predictive performance of sample size, number of features, true model sparsity, signal-to-noise ratio, and feature correlation, in situations where the number of covariates is usually much larger than sample size. Analysis of data sets containing tens of thousands of features but only a few hundred samples is nowadays routine in computational biology, where “omics” features such as gene expression, copy number variation and sequence data are frequently used in the predictive modeling of complex phenotypes such as anticancer drug response. The penalized regression approaches investigated in this study are popular choices in this setting and our simulations corroborate well established results concerning the conditions under which each one of these methods is expected to perform best while providing several novel insights.

## Introduction

Computational biology thrives on a continuous flux of newly proposed algorithms. Methodological developments to solve new problems or improve well established algorithms lie at the heart of the field. Nonetheless, we observe a serious lack in rigorous methodology to objectively and systematically evaluate the performance of competing algorithms. Simulation studies are frequently used to show that a particular method outperforms another. In this context, simulation studies usually involve the generation of a large number of synthetic data sets followed by application and performance comparison of competing methods in each one of the simulated data sets. In principle, this strategy can be used to determine the specific conditions under which a given method outperforms a competing one, and can help guide a user to select an appropriate method based on characteristics of the data. However, in practice, simulation studies often fail to incorporate basic principles of design recommended in the planing of experiments.

In this paper we advocate the use of sound experimental design principles when outlining a simulation study. We adapt well established design techniques, originally developed in the context of physical [1] and computer experiments [2], [3], to simulation studies. As we explain in the detail in the Methods section, a simulation experiment represents a middle ground between computer and physical experiments, and requires the adoption of design techniques from both fields. We denote the Design Of Simulation Experiments by DOSE. We illustrate an application of DOSE to a large scale simulation study comparing ridge [4], lasso [5], and elastic-net [6] regression in situations where the number of features, 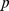, is larger than the number of samples, 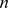.

There are two main motivations for this particular choice of methods. First, predictive modeling in the “large p, small n” setting [7] is an important practical problem in computational biology, with relevant applications in the pharmacogenomics field, where genomic features such as from gene expression, copy number variation, and sequence data have been used, for example, in the predictive modeling of anticancer drug sensitivity [8], [9]. The availability of data sets with large numbers of variables but comparatively small sample sizes has increased the interest in penalized regression models as tools for prediction and variable selection. Standard approaches such as ridge-regression and lasso are commonly used in the analysis of such data sets, and the development of novel approaches, such as elastic-net, have been motivated by applications in the genomic sciences, were the “large 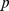, small 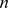
” paradigm is routine.

Second, while these methods are widely used in practice, and their behavior under different conditions is relatively well understood (for instance, the predictive performance of lasso is expected to be better than of ridge-regression in sparse situations, while the reverse is true when the true model is saturated), simulation studies comparing their performance have been limited, focusing on a small number of variables [5], [6]. These characteristics make comparison of regularized regression methods particularly well suited for illustrating DOSE, since we would expect our designed experiment to be able to detect well known behaviors, while also providing novel insights.

As an example of a limited, yet typical, simulation study, consider a fictitious experiment whose objective is to compare the predictive ability of two penalized regression methods, 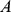 and 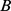. Suppose that method 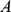 has some theoretical properties that suggest it should outperform method 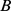 in sparse situations. To empirically test this hypothesis, a researcher designs two simulation experiments. In the first, she/he generates data from 50 correlated covariates and a single response variable associated with all covariates. In the second, the researcher again simulates data from 50 correlated covariates, but with the response variable affected by only 10 covariates. In both simulation studies, he/she fixes the values of the non-zero regression coefficients to 2, sets the residual variance to 1, and generates the covariates from a multivariate normal distribution with mean 0 and correlation matrix with entries given by 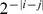, where 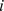 and 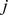 indexes the rows and columns, respectively. For each simulation experiment, she/he generates 100 separate training and test data sets of sample size 300. The researcher optimizes the method's tuning parameters in the training set using 10-fold cross-validation, and evaluates the prediction mean squared error (MSE) using the test set. Finally, suppose that, as a matter of fact, method 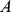 outperformed method 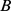 in the sparse setting, whereas the reverse holds in the saturated setting.

No doubt the above simulation result provides some evidence that method 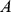 outperforms method 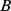 in sparse situations, but can the researcher claim that this simulation demonstrates that method 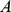 works better than 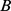 in sparse conditions? In this paper, we argue he/she can not. Because the researcher tested only single, fixed values for the correlation structure among covariates, sample size, residual error, and non-zero regression coefficients, it is not possible to make claims about the relative performance of A and B for parameter regimes not tested in the simulation study. All the researcher can say is that for that particular choice of sample size, signal-to-noise ratio, etc, method 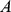 outperforms 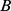 in sparse settings. It is possible the researcher could have observed a different result if she/he had chosen a different combination of simulation parameter values. In other words, the effect of sparsity on the predictive performance, as measured by the difference in MSE of methods 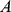 and 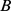, cannot be teased apart from the effects of the other simulation parameters.

To circumvent this problem, we: (i) select simulation parameter values spread as uniformly as possible over the entire parameter space (so that our simulations gather information about all portions of the parameter space); and (ii) generate each simulated data set with a unique combination of simulation parameters. In other words, we adopt a space filling design [2] in our simulations (see Methods section for details). Note that by spreading out the parameter values homogeneously across the parameter space, a space filling design achieves near orthogonality and keeps confounding to a minimum.

In the next section we present the results from our simulation study. See the Methods section for: (i) a brief background on the penalized regression methods studied in this paper; (ii) a comparison of the similarities and differences between computer and simulation experiments; (iii) a description of the space filling experimental design and stochastic data generation process used to generate the simulated data; (iv) details on model fitting and performance evaluation; and (v) a description of the statistical tools used in the analysis of simulation results.

## Results

In order to investigate the absolute and relative predictive ability of penalized regression methods we designed a simulation study focusing on the effects of five distinct simulation parameters, namely: (i) sample size, 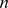; (ii) number of covariates, 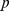; (iii) the saturation parameter, 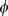, that specifies the probability that a covariate enters the regression model; (iv) signal-to-noise ratio, 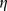, defined as the average of the absolute values of the non-zero regression coefficients divided by the residual variance; and (v) correlation, 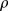, controlling the correlation structure of random blocks of covariates. We concentrate our simulations on situations where the number of covariates (up to 40,000) is usually much larger than the sample size (up to 1,000), since this is usually the case in genomic applications. To the best of our knowledge the present study represents the largest and most systematic simulation experiment comparing regularized regression approaches.

**Figure 1:**
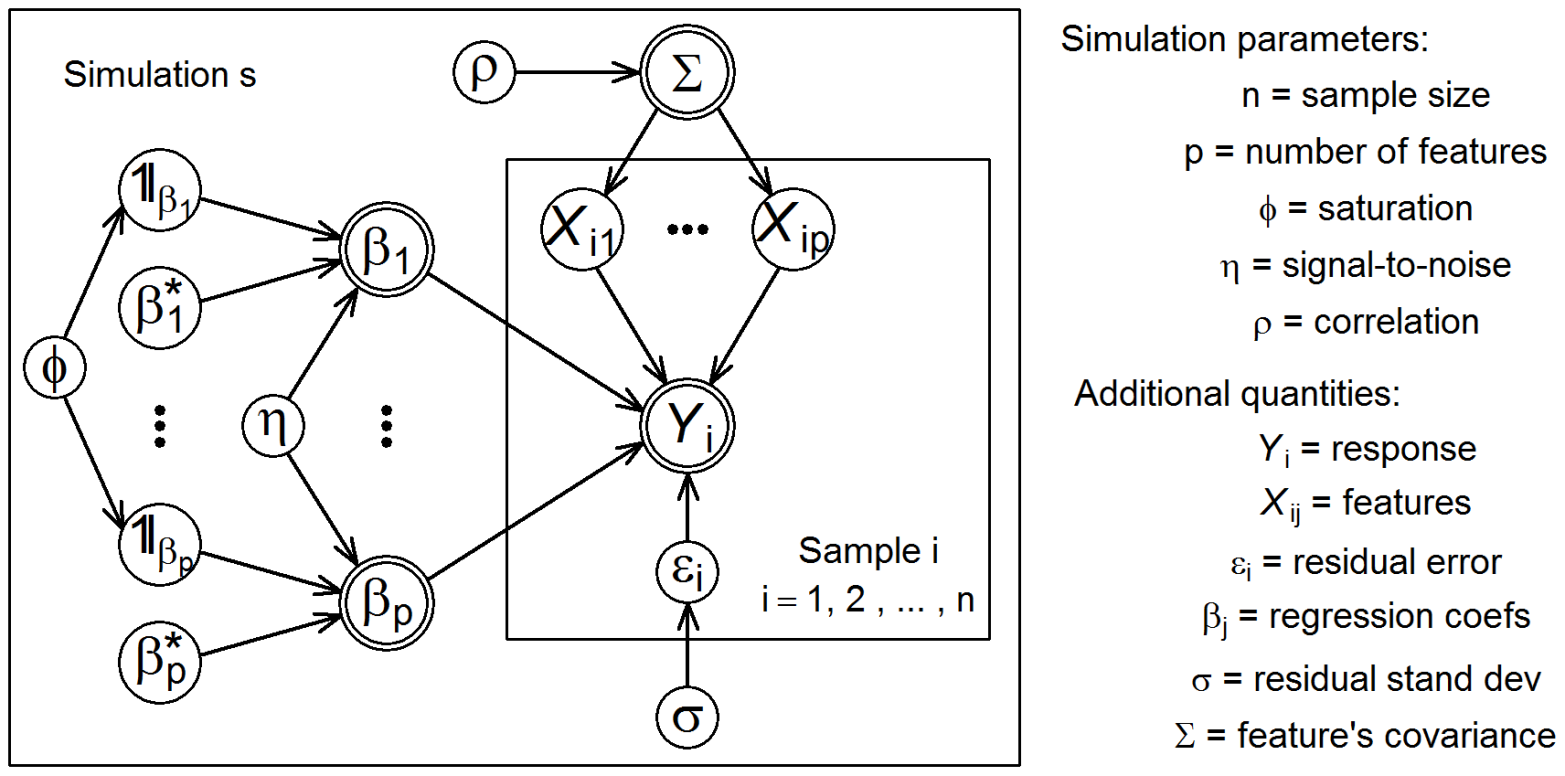
Plate representation of the stochastic data generation process employed in the generation of the simulation study data. Circles represent stochastic variables. Double circles represent functions of the stochastic variables. For each simulation, 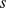, we generate 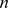 samples 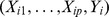, 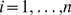. For each sample, 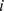, we simulate 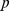 correlated features, 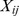, conforming to a covariance matrix 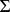 parameterized according to the correlation parameter, 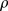. The response, 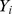, is generated from a linear regression model with residual variance, 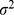, set to 1. The indicator variables, 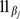, control which features influence the response, according to the inclusion probability 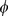 (which controls the saturation of the model). The regression coefficients, 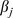, are obtained by re-scaling the 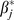 values, in order to control the signal-to-noise ratio, 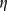. See the Methods section for details, including the distributional assumptions associated with these quantities.

In the Methods section we provide details on simulation parameter ranges and the stochastic data generation process employed in the generation of the simulated data sets. Figure 1 presents a graphical model representation and overview of the data generation process. In total, we generated 10,000 distinct simulated data sets according to a space filling design.

**Figure 2:**
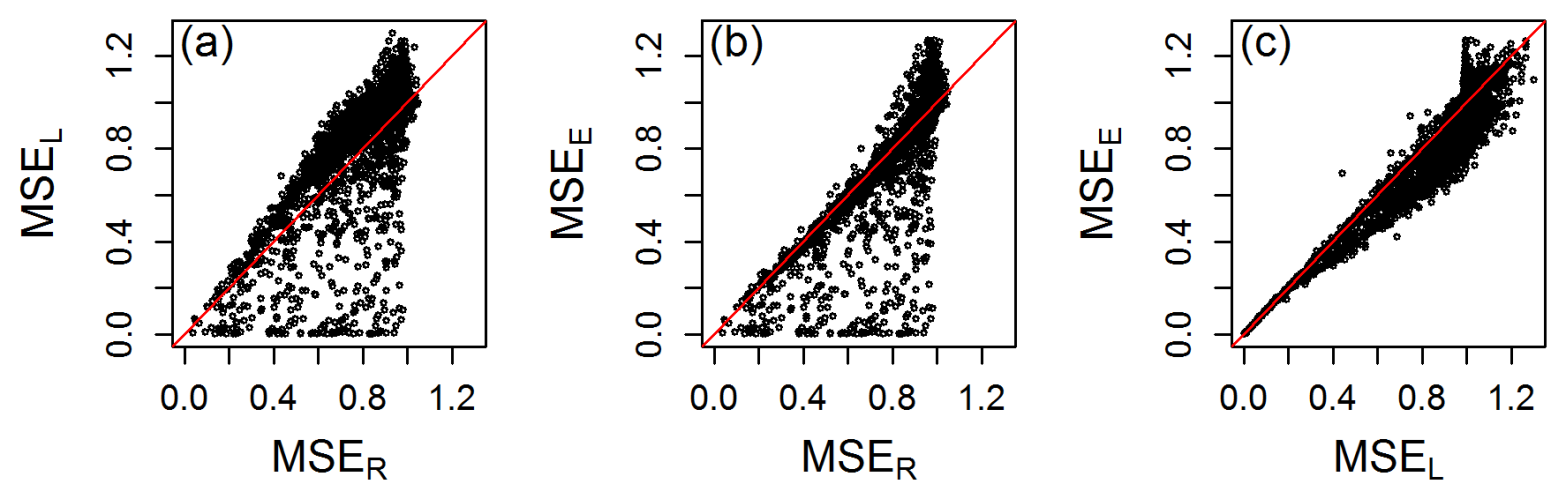
Scatter plots comparing the MSE scores produced by ridge-regression, lasso, and elastic-net. Panel a shows the comparison of ridge-regression vs lasso, panel b compares ridge-regression vs elastic-net, and panel c compares lasso vs elastic-net.

Figure 2 presents scatter plots of the MSEs of ridge-regression, lasso and elastic-net. Overall, ridge showed smaller MSE than lasso in 90.46% of the simulations (panel a). This result is not unexpected since the maximum number of covariates that can be selected by lasso cannot be larger than the sample size, and in 95.24% of the simulations the true model contained more than *n* covariates. Most of the cases were lasso outperformed ridge-regression (below diagonal dots) correspond to the simulations where the sample size was larger than the number of covariates in the true model; that is, where the true model was sparse (see Figure S1a in the Supplement for further details).

The comparative performance of elastic-net and ridge-regression was quite balanced (panel b), with elastic-net outperforming ridge in 52.74% of the simulations, and most of the simulations showing close MSE values (note the concentration of points along the diagonal red curve). The simulations where elastic-net performed considerably better than ridge, i.e., the points dispersed to the lower right of the diagonal, correspond to sparse true models (see Figure S1b for details).

Elastic-net outperformed lasso in 86.80% of the simulations (panel c). Again, this is not an unexpected result since, as described above, the performance of lasso is compromised when the number of covariates in the true model is larger than sample size, whereas elastic net can select more than *n* covariates [6]. In the next subsections we investigate in detail the absolute and relative predictive performances of these three penalized regression methods.

### Ridge-regression

**Figure 3:**
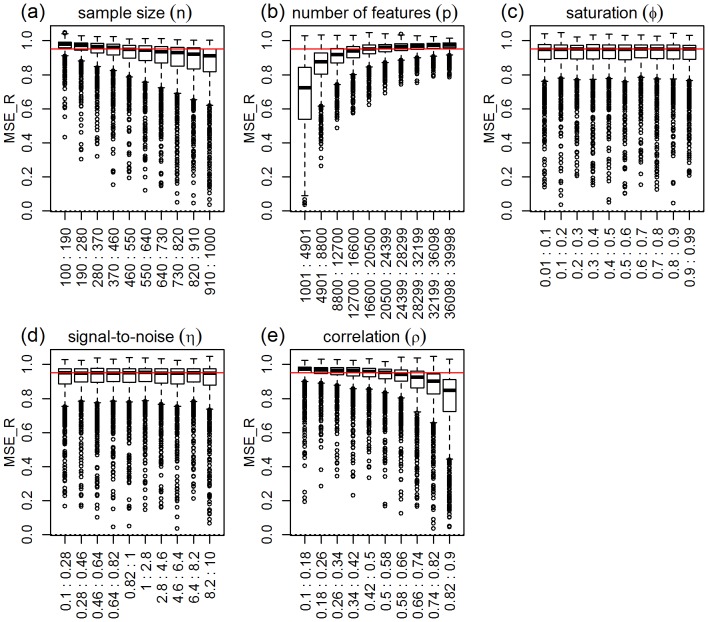
Distributions of the absolute performance response for ridge-regression, across 10 equally spaced bins of the parameters ranges. The x-axis show the parameter ranges comprised by each of the 10 bins. The y-axis shows the absolute performance response 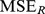. The red horizontal line represents the median of the response distribution. The dotted line is set at zero.

**Figure 4:**
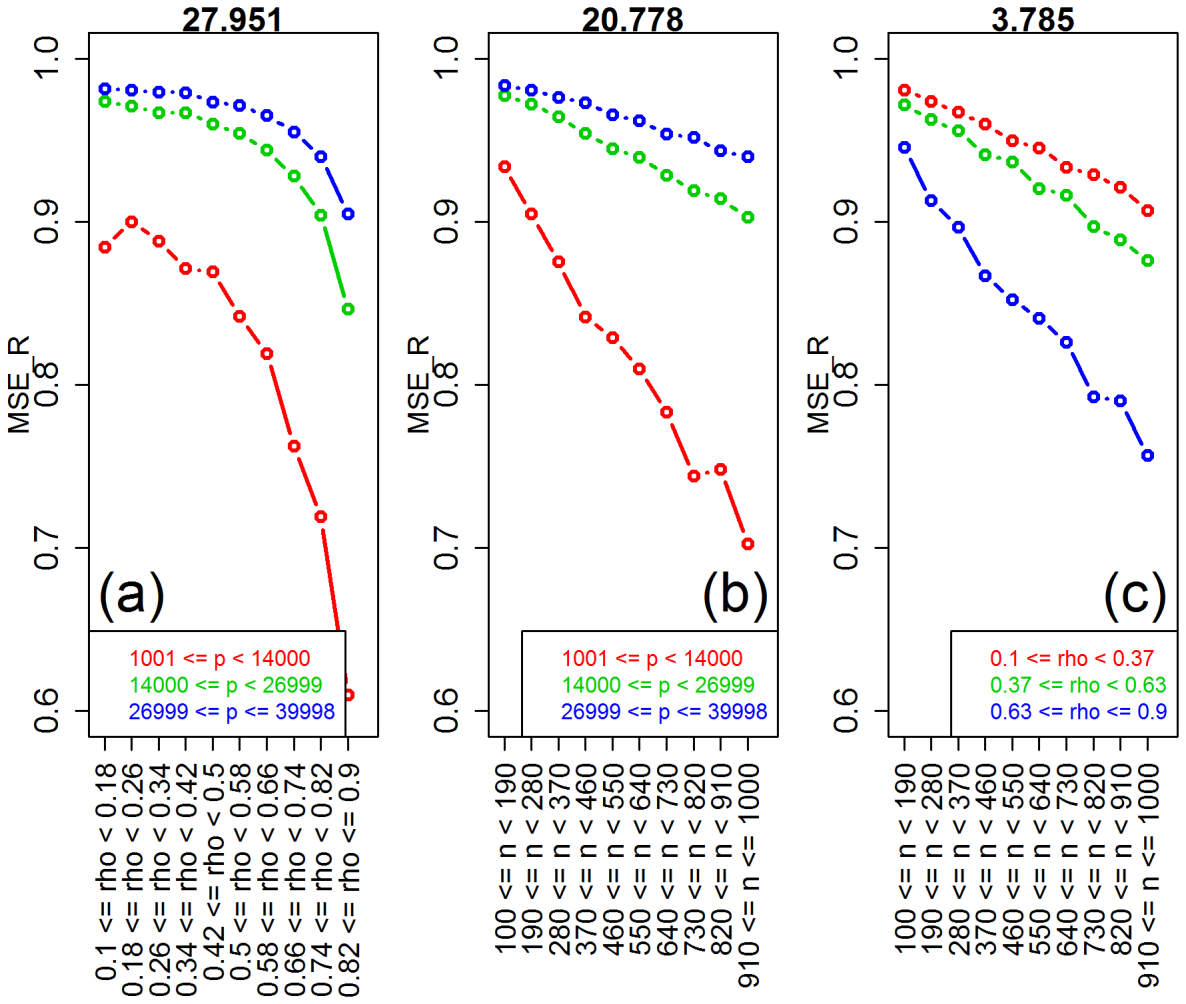
Interaction plots for ridge-regression. The values of the interaction test statistics are shown on the top of the figures.

In this section, we investigate the effect of the simulation parameters on the mean squared error of the ridge model (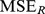). Figure 3 presents the response distributions for each one of the five simulation parameters across their respective ranges (represented by 10 equally spaced bins). The red horizontal line represents the median of the response distribution. Inspection of the plots shows clear shifts in location and/or spread for the number of features, correlation, and sample size parameters, but practically constant distributions across the binned groups for the saturation parameter and (slightly less so) for the signal-to-noise parameter. Permutation tests for the equality of group distributions null hypothesis (using distance component analysis [10] - see the “Data analysis” subsection on Methods for details) presented in Table 1, confirm that the group differences are highly significant (p-value 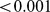) for the number of features, correlation, and sample size parameters (note the high values of the observed test statistics), but non-significant for saturation, and marginally significant for the signal-to-noise parameter. Table 1 also shows highly significant interactions for number of features vs correlation, sample size vs number of features, and sample size vs correlation. Figure 4 shows the respective interaction plots.

**Table 1:** Ridge-regression.

par.	obs. stat.	p-value
sample size ( 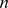 )	76.980	<0.001
number of features ( 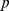 )	450.212	<0.001
saturation ( 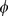 )	0.651	0.939
signal-to-noise ( 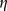 )	1.354	0.098
correlation ( 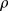 )	141.839	<0.001
sample size vs number of features ( 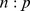 )	20.778	<0.001
sample size vs saturation ( 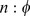 )	0.559	0.998
sample size vs signal-to-noise ( 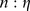 )	0.593	0.998
sample size vs correlation ( 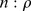 )	3.785	<0.001
number of features vs saturation ( 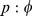 )	0.553	1.000
number of features vs signal-to-noise ( 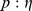 )	1.139	0.207
number of features vs correlation ( 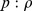 )	27.951	<0.001
saturation vs signal-to-noise ( 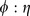 )	0.760	0.952
saturation vs correlation ( 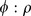 )	0.677	0.980
signal-to-noise vs correlation ( 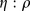 )	0.727	0.965

Permutation tests for equality of the group distributions using distance components analysis (lines 2 to 6), and permutation F-tests for the presence of 2-by-2 interactions (lines 7 to 16). Results based on 999 permutations.

As expected, Figure 3 shows improvement in predictive performance as the sample size increases, the number of features decreases and the amount of correlation increases (panels a, b, and e, respectively). Furthermore, the interaction plots in Figure 4 show synergistic effects of these parameters, with larger improvements in performance achieved by combinations of larger sample sizes with smaller number of features and with larger correlation values. Note that the strong decrease in MSE as a function of the amount of correlation among the features is expected since ridge-regression is highly effective in ameliorating multi-collinearity problems (the purpose for which it was originally developed). The lack of influence of the saturation parameter on the predictive performance is expected for ridge-regression, whereas the marginal influence of the signal-to-noise parameter is investigated in more detail in Text S1.

### Lasso

**Figure 5:**
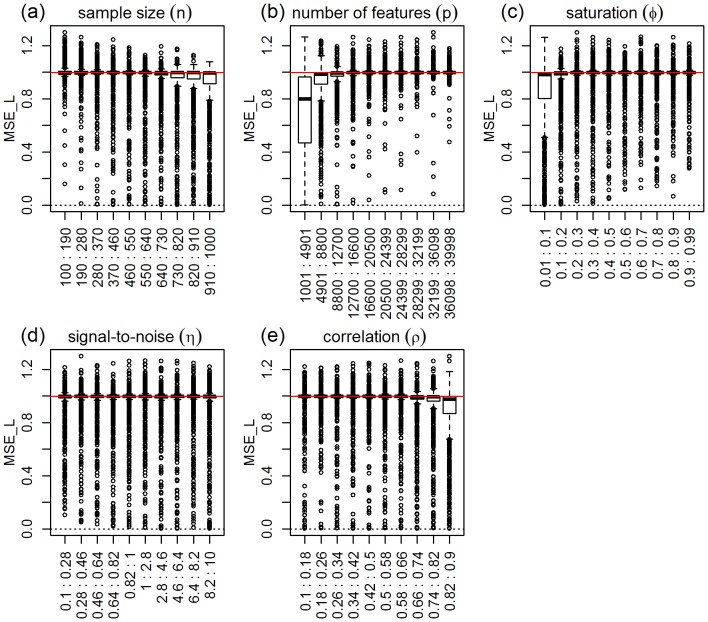
Distributions of the absolute performance response for lasso, across 10 equally spaced bins of the parameters ranges. The x-axis show the parameter ranges comprised by each of the 10 bins. The y-axis shows the absolute performance response 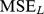. The red horizontal line represents the median of the response distribution. The dotted line is set at zero.

**Figure 6:**
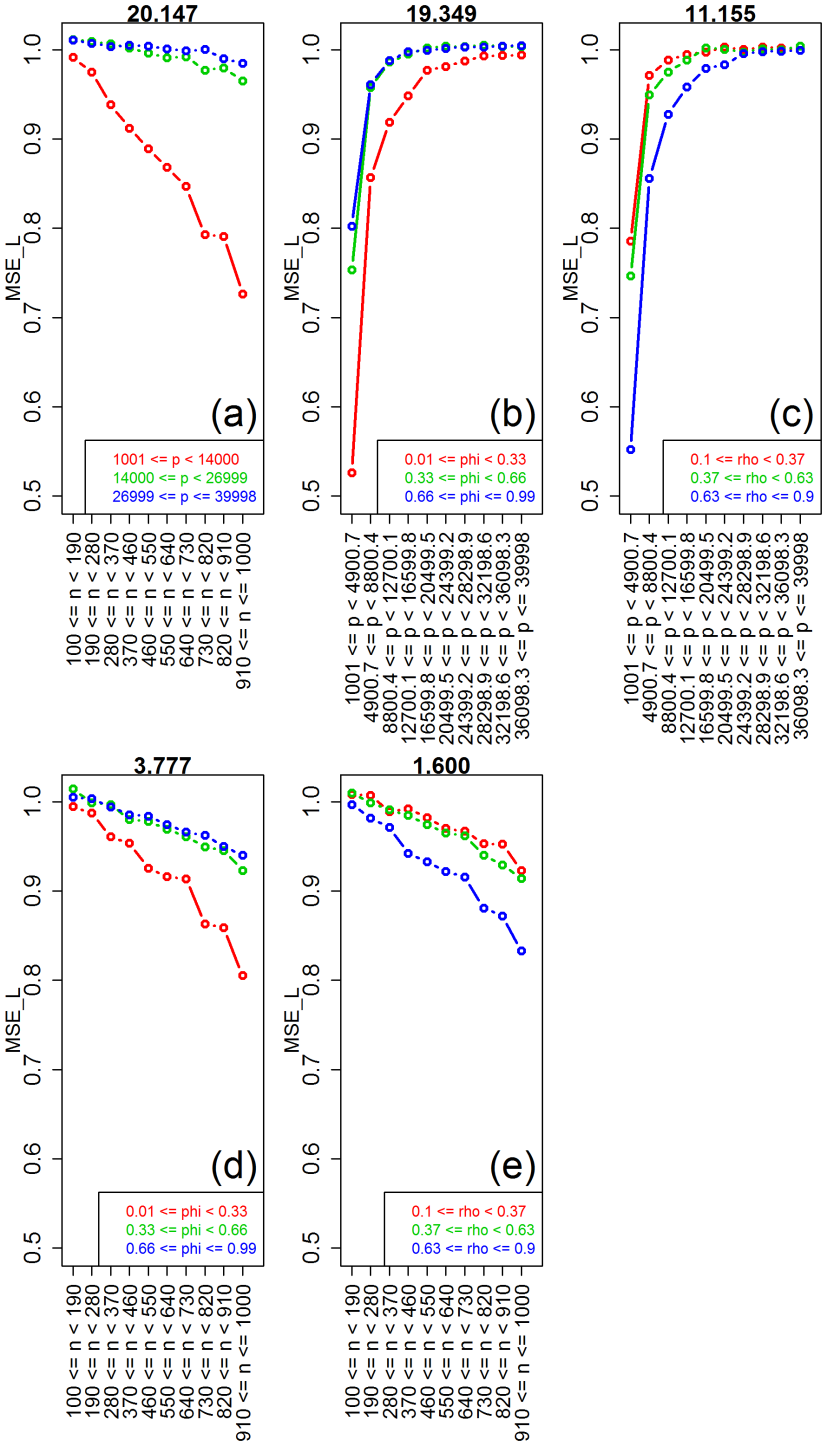
Interaction plots for lasso. The values of the interaction test statistics are shown on the top of the figures.

For lasso, Figure 5 shows strong shifts in location and/or spread for all parameters, except for the signal-to-noise. The permutation tests (Table 2) confirms these results, and shows that even the much weaker group differences for the signal-to-noise parameter are statistically significant. Highly significant interaction terms included: sample size vs number of features, number of features vs saturation, number of features vs correlation, sample size vs saturation, and sample size vs correlation. Figure 6 shows the respective interaction plots.

**Table 2:** Lasso.

par.	obs. stat.	p-value
sample size ( 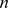 )	38.094	<0.001
number of features ( 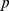 )	328.013	<0.001
saturation ( 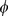 )	50.773	<0.001
signal-to-noise ( 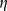 )	2.379	<0.001
correlation ( 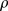 )	34.313	<0.001
sample size vs number of features ( 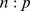 )	20.147	<0.001
sample size vs saturation ( 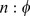 )	3.777	<0.001
sample size vs signal-to-noise ( 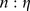 )	0.864	0.813
sample size vs correlation ( 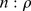 )	1.600	<0.001
number of features vs saturation ( 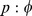 )	19.349	<0.001
number of features vs signal-to-noise ( 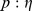 )	1.222	0.092
number of features vs correlation ( 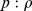 )	11.155	<0.001
saturation vs signal-to-noise ( 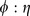 )	0.963	0.581
saturation vs correlation ( 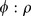 )	0.569	0.999
signal-to-noise vs correlation ( 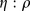 )	0.620	0.997

Permutation tests for equality of the group distributions using distance components analysis (lines 2 to 6), and permutation F-tests for the presence of 2-by-2 interactions (lines 7 to 16). Results based on 999 permutations.

Figure 5 shows improvement in predictive performance as the sample size increases, the number of features decreases, the saturation decreases, and the amount of correlation increases (panels a, b, c, and e, respectively). Once again, the interaction plots in Figure 6 show synergistic effects of these parameters, with larger improvements in performance achieved by combinations of larger sample sizes with smaller number of features, smaller saturation and with larger correlation values. For lasso too, we observe a considerable decrease in MSE as a function of the amount of correlation in the features (although not as strong as in ridge-regression) corroborating empirical observations that although lasso can combat multi-collinearity problems it is not as effective as ridge-regression. On the other hand, and contrary to ridge-regression that is insensitive to the influence of the saturation parameter, we clearly observe an improvement in MSE as a function of decreasing saturation values for the lasso. The marginal influence of the signal-to-noise parameter is again investigated in more detail in Text S1.

### Elastic-net

**Figure 7:**
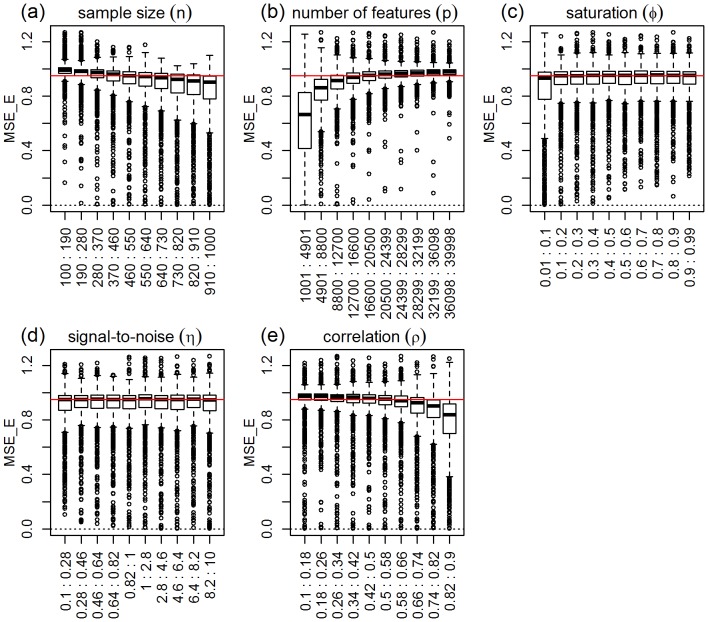
Distributions of the absolute performance response for elastic-net, across 10 equally spaced bins of the parameters ranges. The x-axis show the parameter ranges comprised by each of the 10 bins. The y-axis shows the absolute performance response 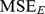. The red horizontal line represents the median of the response distribution. The dotted line is set at zero.

**Figure 8:**
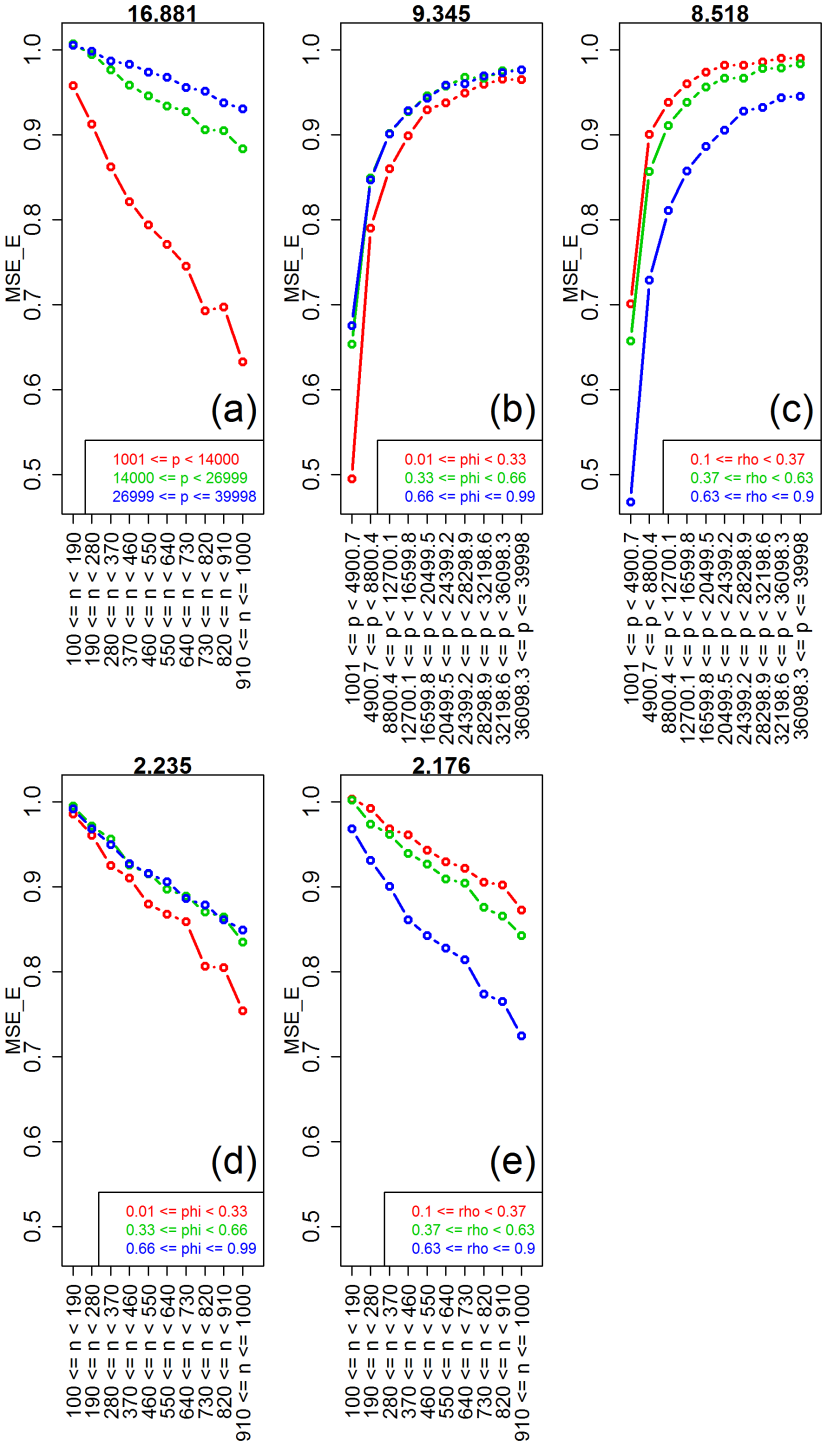
Interaction plots for elastic-net. The values of the interaction test statistics are shown on the top of the figures.

For elastic-net, Figure 7 shows clear shifts in location and/or spread for all parameters, except for the signal-to-noise. Table 3, confirms these results, and shows that even the much weaker group differences for the signal-to-noise parameter are statistically significant. Table 3 also shows highly significant interactions for sample size vs number of features, number of features vs saturation, number of features vs correlation, sample size vs saturation, and sample size vs correlation. Figure 8 shows the respective interaction plots.

**Table 3:** Elastic-net.

par.	obs. stat.	p-value
sample size ( 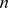 )	92.070	<0.001
number of features ( 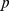 )	393.980	<0.001
saturation ( 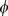 )	18.031	<0.001
signal-to-noise ( 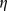 )	1.882	0.006
correlation ( 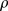 )	97.958	<0.001
sample size vs number of features ( 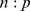 )	16.881	<0.001
sample size vs saturation ( 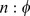 )	2.235	<0.001
sample size vs signal-to-noise ( 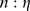 )	0.776	0.927
sample size vs correlation ( 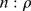 )	2.176	<0.001
number of features vs saturation ( 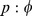 )	9.345	<0.001
number of features vs signal-to-noise ( 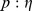 )	1.245	0.076
number of features vs correlation ( 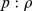 )	8.518	<0.001
saturation vs signal-to-noise ( 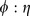 )	1.014	0.445
saturation vs correlation ( 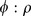 )	0.605	0.997
signal-to-noise vs correlation ( 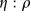 )	0.665	0.994

Permutation tests for equality of the group distributions using distance components analysis (lines 2 to 6), and permutation F-tests for the presence of 2-by-2 interactions (lines 7 to 16). Results based on 999 permutations.

As expected, Figure 7 shows improvement in predictive performance as the sample size increases, the number of features decreases, the saturation decreases, and the amount of correlation increases (panels a, b, c, and e, respectively). Furthermore, the interaction plots in Figure 8 show, once again, synergistic effects of these parameters with larger improvements in performance achieved by combinations of larger sample sizes with smaller number of features, smaller saturation and with larger correlation values. For elastic-net, we observe a strong decrease in MSE as a function of the amount of correlation in the features (comparable to ridge-regression) corroborating empirical observations that elastic-net can be as efficient as ridge-regression in the combat multi-collinearity problems. Furthermore, and similarly to lasso, we clearly observe improvement in MSE as a function of decreasing saturation values for the elastic-net. The marginal influence of the signal-to-noise parameter is investigated in detail in Text S1.

### Ridge-regression versus lasso

**Figure 9:**
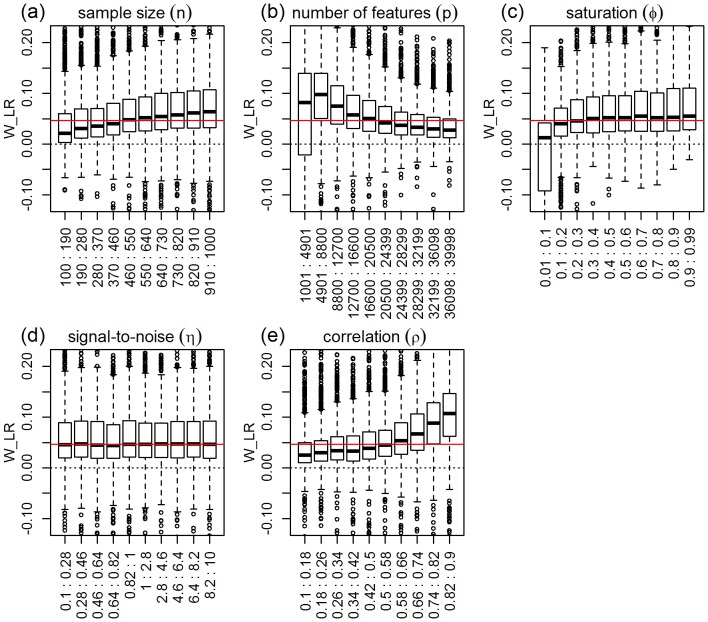
Distributions of the relative performance response in the ridge-regression vs lasso comparison, across 10 equally spaced bins of the parameters ranges. The x-axis show the parameter ranges comprised by each of the 10 bins. The y-axis shows the relative performance response 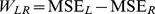. The red horizontal line represents the median of the response distribution. The dotted line is set at zero.

**Figure 10:**
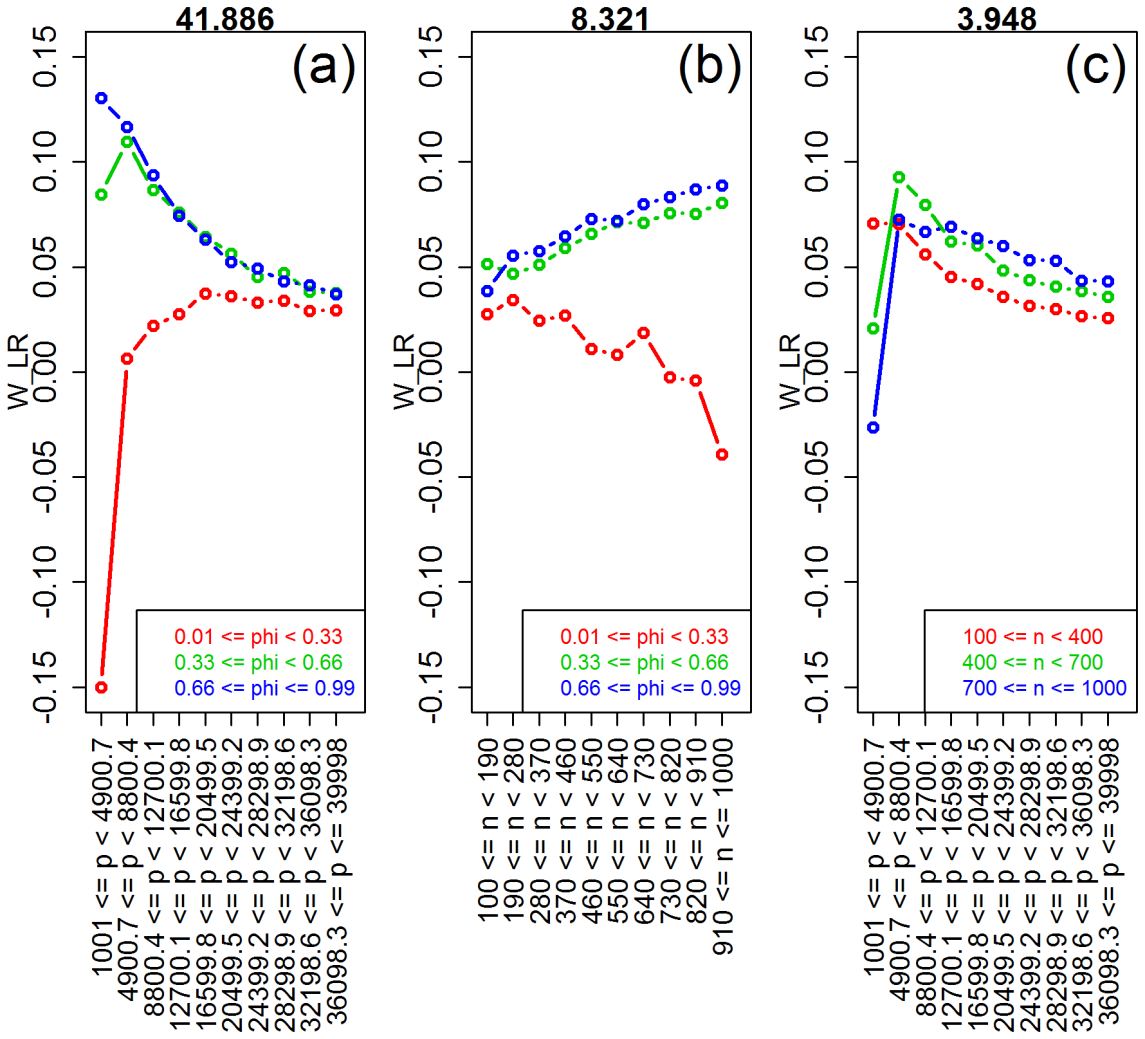
Interaction plots for the ridge-regression vs lasso comparison. The values of the interaction test statistics are shown on the top of the figures.

In order to compare the predictive performance of ridge-regression against lasso we defined the response as 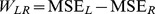. Note that positive values of the response represent the simulations where ridge-regression outperforms lasso, and vice-versa. Figure 9 presents the response distributions for each one of the five simulation parameters. Inspection of the plots shows clear shifts in location and/or spread for the number of features, correlation, saturation, and sample size, but practically constant distribution across the bins for the signal-to-noise parameter. Permutation tests for the equality of group distributions null hypothesis, presented in Table 4, confirm that the group differences are highly significant (p-value 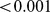) for the number of features, correlation, saturation, and sample size parameters (note the high values of the observed test statistics), but non-significant for signal-to-noise. Table 4 also shows highly significant interactions for number of features vs saturation, sample size vs saturation, and sample size vs number of features. Figure 10 shows the respective interaction plots.

**Table 4:** Ridge-regression vs lasso.

par.	obs. stat.	p-value
sample size ( 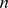 )	28.003	<0.001
number of features ( 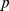 )	99.312	<0.001
saturation ( 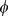 )	78.062	<0.001
signal-to-noise ( 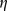 )	0.949	0.541
correlation ( 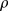 )	85.745	<0.001
sample size vs number of features ( 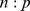 )	3.948	<0.001
sample size vs saturation ( 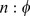 )	8.321	<0.001
sample size vs signal-to-noise ( 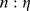 )	1.002	0.482
sample size vs correlation ( 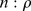 )	0.996	0.490
number of features vs saturation ( 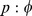 )	41.886	<0.001
number of features vs signal-to-noise ( 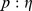 )	1.023	0.426
number of features vs correlation ( 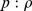 )	1.393	0.017
saturation vs signal-to-noise ( 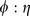 )	1.017	0.443
saturation vs correlation ( 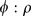 )	0.570	1.000
signal-to-noise vs correlation ( 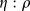 )	0.695	0.986

Permutation tests for equality of the group distributions using distance components analysis (lines 2 to 6), and permutation F-tests for the presence of 2-by-2 interactions (lines 7 to 16), in the comparison of ridge-regression vs lasso. Results based on 999 permutations.

Comparison of ridge-regression against lasso corroborate two well known results, namely: (i) that lasso outperforms ridge when the true model is sparse, whereas the converse holds true for saturated models (Figure 9c); and (ii) for highly correlated features, ridge tends to dominate lasso in terms of predictive performance (Figure 9e). More interestingly, our simulations also detected a couple of less appreciated patterns. First, Figure 9a shows that the average advantage of ridge-regression over lasso tends to increase as the sample size gets larger. Nonetheless, the interaction plots in Figures 10b and c show that this advantage is larger in moderate to highly saturated models, but that lasso tends to outperform ridge-regression when sample size is large, but number of features and saturation are small. Second, Figure 9b shows an interesting pattern for the number of features, where the advantage of ridge-regression over lasso tends to increase at first, and then decreases as the number of covariates increases further. Figure 10a provides an explanation for this curious trend. Lasso is clearly better for small number of features if the saturation is also small, but ridge is better if the saturation is moderate to large. The advantage of ridge decreases with the number of features (for moderate or large saturation). With small saturation, increasing the number of features makes ridge more competitive, since at some point the number of features entering the model (i.e. 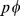, on average) become larger than the sample size.

### Ridge-regression versus elastic-net

**Figure 11:**
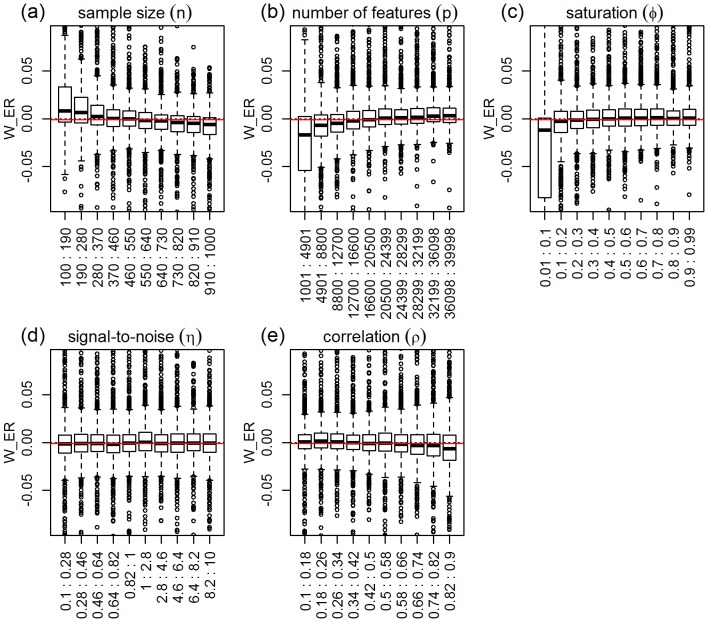
Distributions of the relative performance response in the ridge-regression vs elastic-net comparison, across 10 equally spaced bins of the parameters ranges. The x-axis show the parameter ranges comprised by each of the 10 bins. The y-axis shows the relative performance response 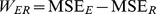. The red horizontal line represents the median of the response distribution. The dotted line is set at zero.

**Figure 12:**
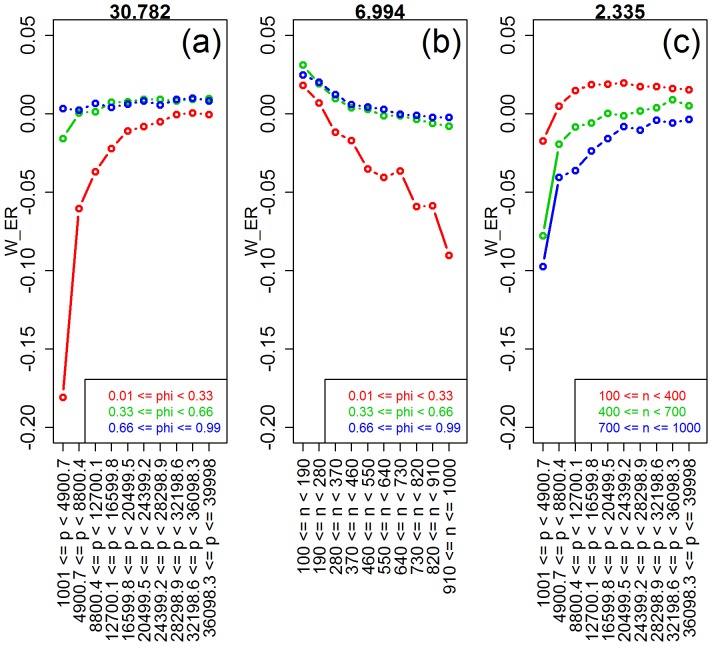
Interaction plots for the ridge-regression vs elastic-net comparison. The values of the interaction test statistics are shown on the top of the figures.

For the comparison of ridge-regression against elastic-net we defined the response as 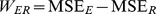, so that positive values show the simulations where ridge-regression outperforms elastic-net, and vice-versa. Figure 11 shows clear shifts in location and spread of the boxplots for saturation, number of features, sample size, and correlation, but considerably constant distribution for the signal-to-noise parameter. Overall, we see that the predictive performances of ridge and elastic-net tend to be closer to each other than the performances of ridge and lasso (note the small spread of most of the boxplots, and the closeness of the boxplot medians to 0). The permutation tests (Table 5) detected highly significant differences in group distributions for saturation, number of features, sample size, and correlation, and marginally significant differences for signal-to-noise. Highly significant interaction terms included: number of features vs saturation, sample size vs saturation, and sample size vs number of features. Figure 12 shows the respective interaction plots.

**Table 5:** Ridge-regression vs elastic-net.

par.	obs. stat.	p-value
sample size ( 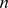 )	42.231	<0.001
number of features ( 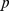 )	61.468	<0.001
saturation ( 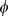 )	82.652	<0.001
signal-to-noise ( 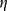 )	1.515	0.023
correlation ( 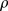 )	6.099	<0.001
sample size vs number of features ( 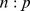 )	2.335	<0.001
sample size vs saturation ( 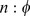 )	6.994	<0.001
sample size vs signal-to-noise ( 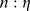 )	1.049	0.365
sample size vs correlation ( 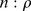 )	0.675	0.996
number of features vs saturation ( 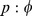 )	30.782	<0.001
number of features vs signal-to-noise ( 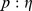 )	1.239	0.075
number of features vs correlation ( 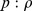 )	1.685	<0.001
saturation vs signal-to-noise ( 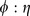 )	1.417	0.009
saturation vs correlation ( 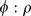 )	0.539	1.000
signal-to-noise vs correlation ( 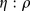 )	0.739	0.967

Permutation tests for equality of the group distributions using distance components analysis (lines 2 to 6), and permutation F-tests for the presence of 2-by-2 interactions (lines 7 to 16), in the comparison of ridge-regression vs elastic-net. Results based on 999 permutations.

Comparison of ridge-regression against elastic-net corroborates the well known result that elastic-net tends to show much better performance than ridge-regression when the true model is sparse, while these methods tend to be comparable for saturated models (Figure 11c). Novel insights uncovered by our simulations include that: (i) ridge tends to outperform elastic-net when sample size is small, but the reverse is true for larger sample sizes (Figure 11a). Furthermore, the interaction plots in Figure 12 show that the better performance of elastic-net is accentuated when sample size is large but number of features and saturation are small; (ii) elastic-net tends to outperform ridge when number of features is small, but both methods tend to become comparable (with ridge being slightly better) for larger number of features (Figure 11b). This pattern is explained by a strong interaction between number of features and saturation (Figure 12a), which shows that the elastic-net performs much better than ridge when the number of features is small and the true model is sparse; and (iii) elastic-net tends to perform slightly better than ridge when the covariates are highly correlated (Figure 11e).

### Lasso versus elastic-net

**Figure 13:**
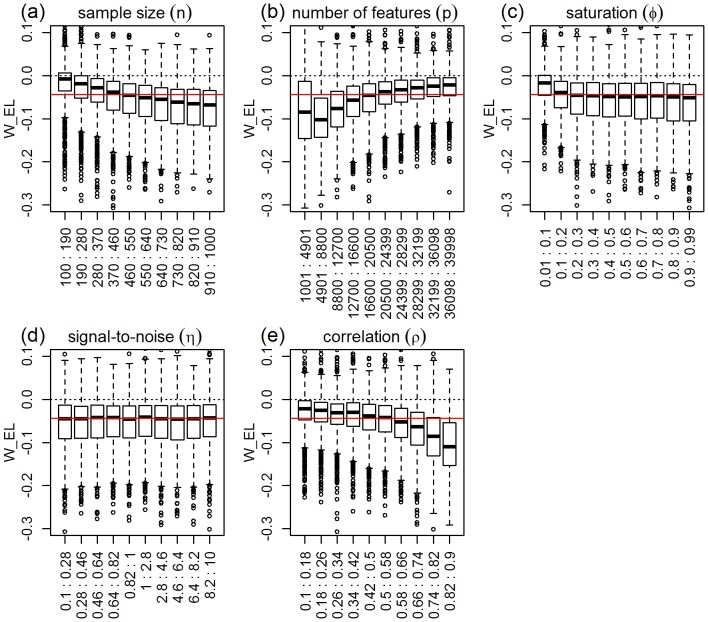
Distributions of the relative performance response in the elastic-net vs lasso comparison, across 10 equally spaced bins of the parameters ranges. The x-axis show the parameter ranges comprised by each of the 10 bins. The y-axis shows the relative performance response 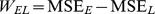. The red horizontal line represents the median of the response distribution. The dotted line is set at zero.

For the comparison of lasso against elastic-net we defined the response as 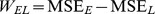. Hence, positive values of the response show the simulations where lasso outperforms elastic-net, and vice-versa. Figure 13 shows clear distribution differences for the number of features, correlation, sample size, and saturation parameters, but practically no differences for signal-to-noise. Table 6 corroborates these findings showing a non-significant p-value for signal-to-noise, but highly significant results for all other parameters. The permutation tests also detected highly significant interactions for: number of features vs saturation, sample size vs number of features, number of features vs correlation, sample size vs saturation, and sample size vs correlation.

**Table 6:** Lasso vs elastic-net.

par.	obs. stat.	p-value
sample size ( 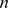 )	78.683	<0.001
number of features ( 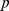 )	123.014	<0.001
saturation ( 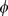 )	26.688	<0.001
signal-to-noise ( 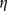 )	0.804	0.781
correlation ( 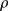 )	115.291	<0.001
sample size vs number of features ( 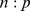 )	6.341	<0.001
sample size vs saturation ( 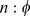 )	2.212	<0.001
sample size vs signal-to-noise ( 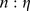 )	0.771	0.932
sample size vs correlation ( 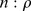 )	1.821	<0.001
number of features vs saturation ( 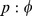 )	9.788	<0.001
number of features vs signal-to-noise ( 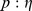 )	0.652	0.991
number of features vs correlation ( 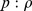 )	6.168	<0.001
saturation vs signal-to-noise ( 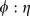 )	0.735	0.971
saturation vs correlation ( 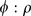 )	1.233	0.084
signal-to-noise vs correlation ( 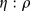 )	0.544	0.999

Permutation tests for equality of the group distributions using distance components analysis (lines 2 to 6), and permutation F-tests for the presence of 2-by-2 interactions (lines 7 to 16), in the comparison of lasso vs elastic-net. Results based on 999 permutations.

**Figure 14:**
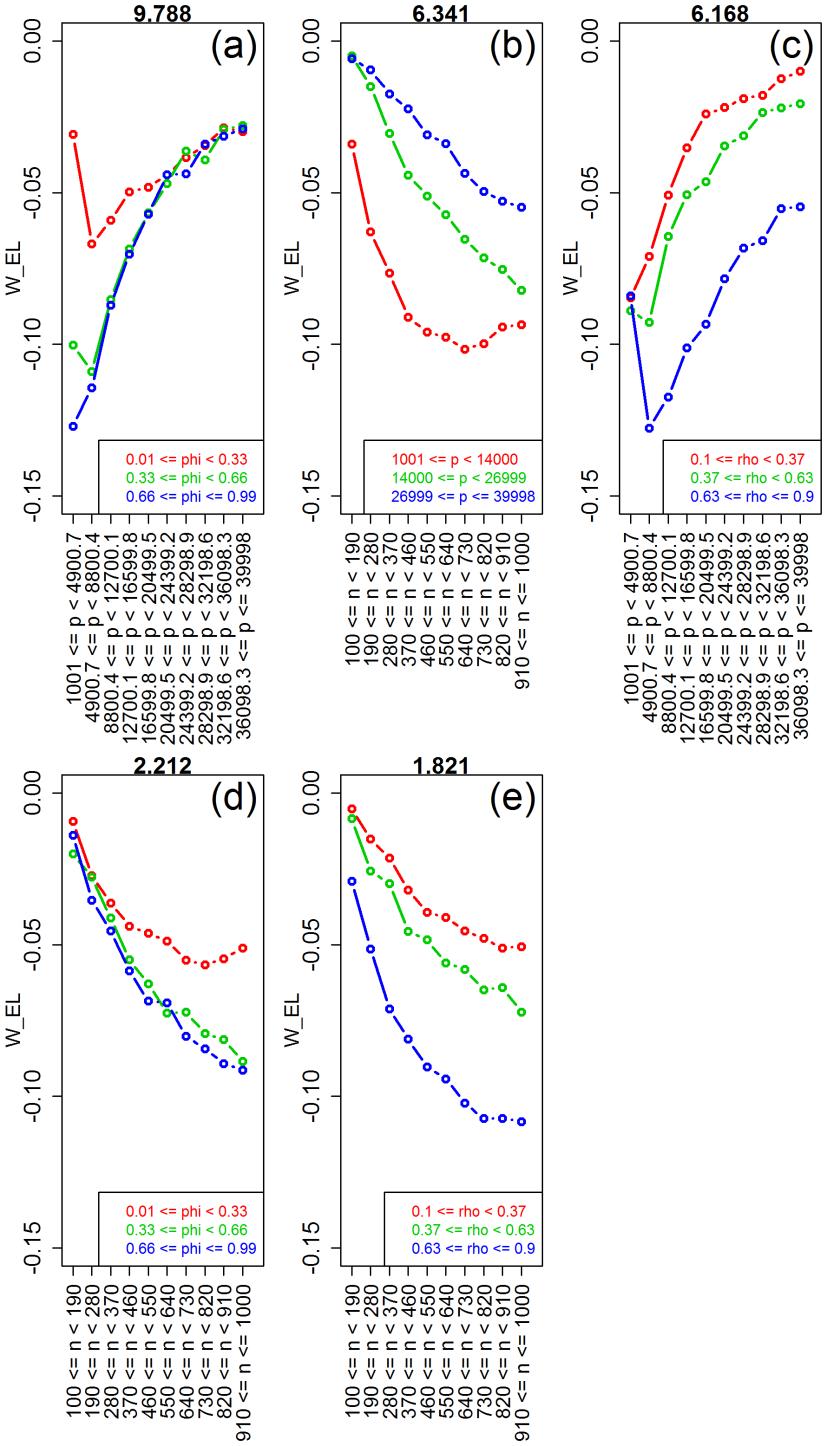
Interaction plots for the lasso vs elastic-net comparison. The values of the interaction test statistics are shown on the top of the figures.

Comparison of lasso versus elastic-net also corroborates the well established results that: (i) lasso and elastic-net show comparable performances when the true model is sparse, whereas elastic-net outperforms lasso when the true model is saturated; and (ii) elastic-net outperforms lasso when the covariates are highly correlated. Furthermore, our simulations generated a couple of new insights. First, the advantage of elastic-net over lasso increases as the sample size gets larger (Figure 13a). Figures 14b, d and e, show that this advantage of elastic-net is more accentuated: for smaller number of features (red curve in Figure 14b); for moderate to larger saturations (green and blue curves in Figure 14d); and for larger correlations (blue curve in Figure 14e). Together, these results explain the larger spread of the boxplots on Figure 13a as sample size gets larger. Second, Figure 13b shows that, relative to number of features, the advantage of elastic-net tends to increase at first, but then starts to decrease as the number of features increases. Figures 14a and 14c provide an explanation for this curious trend. Figure 14a shows that elastic-net is clearly better than lasso for smaller number of features, if saturation is moderate or large, but lasso becomes more competitive when saturation is small, and that the advantage of elastic-net decreases with the number of features. Figure 14c shows that, when the correlation is high, the advantage of elastic-net tends to increase rapidly (as the number of features reaches approximately 8,800), before it starts to decrease with increasing number of features.

## Discussion

In this paper we propose running simulation studies as designed experiments. We argue that, when comparing the performance of alternative methods in a simulation study, it is important to use well established design principles and follow best practices in planning of experiments, such as choosing relevant simulation parameters, adopting realistic ranges of parameter values, and making use of pilot studies to improve the design of the actual simulation experiment (see subsection “Choice of simulation parameters and parameter ranges” in Methods for details).

We illustrate the application of DOSE in a large scale simulation study comparing the relative performance of popular penalized regression methods as a function of sample size, number of features, model saturation, signal-to-noise ratio, and strength of correlation between groups of covariates organized in a blocked structure. We restricted our simulations to the case where the number of features is larger than the number of samples, since this is the usual setting in the analysis of real genomic data. Our simulations corroborated all well-established results concerning the conditions under which ridge-regression, lasso, and elastic-net are expected to perform best, but also provided several novel insights, described in the Results section.

In the present work we adopted MSE as the scoring metric since it is widely used in practice and is the metric adopted in the original papers proposing the lasso and elastic-net approaches. Nonetheless, it is important to point out that the results presented in this paper could be metric dependent, as alternative metrics might rank competing models differently. Interesting alternatives to the MSE metric include: concordance correlation coefficient [11], Pearson correlation, and mean absolute error. We point out, however, that an in-depth robustness investigation of our results with respect to alternative scoring metrics is out of the scope of the present paper, and is left as an interesting future research project.

The incorporation of experimental design techniques in simulation studies can be useful in computational biology for three main reasons. (1) First, it provides increased objectivity and thoroughness in the assessment of competing methods/algorithms. (2) Second, it might improve our ability to select existing methods based on characteristics of a given data set. For instance, suppose that a researcher is working in a pharmacogenomics data set, aiming to perform predictive modeling of drug response sensitivity. Suppose further that comparison of ridge-regression and lasso model fits shows a better predictive performance by ridge (as is often the case in pharmacogenomic data sets [12], [13]). Next the researcher needs to decide between ridge-regression and elastic-net. At this point, instead of running elastic-net, a computationally expensive approach which requires performing cross-validation for two tuning parameters, the researcher can guide his/her choice based on readily observable characteristics of the data set, such as, sample size, number of features, and amount of correlation among the features (or assumptions about unobserved characteristics, such as underlying model sparsity). For instance, if her/his data has a small number of samples and a large number of weakly correlated features, our simulations suggest (see panels a, b, and e, on Figure 11 and Figure 12) that ridge-regression is more likely to outperform elastic-net than the converse. (3) Third, the adoption of design techniques improves our ability to demonstrate the strengths of a method under specific conditions. This point is important, since it is unrealistic to expect any given method to outperform its competitors across a large panel of data sets, with diverse characteristics such as different sample sizes, amount of signal, correlation structures, etc. A more realistic goal is to demonstrate the improved performance of the given method under specific conditions, i.e., in a subspace of data set characteristics.

This third point closely resonates with the celebrated No Free Lunch (NFL) theorems [14]–[16], which can be interpreted as a formalization of David Hume's critique of the inductive method in science. More formally, in the context of supervised learning with zero-one loss, Wolpert [15] shows that, if we don't make any assumptions (or, have no prior information) about the target input-output relationship we are trying to learn, than for any two algorithms A and B there are as many targets for which A outperforms B as vice versa. Extensions of NFL theorems for other loss functions are discussed in [16], which provides analogous (although weaker) NFL-type theorems applicable to the quadratic loss function. The NFL theorems address “the mathematical ‘skeleton’ of supervised learning, before the ‘flesh’ of particular priors” (i.e., assumptions) concerning the target input-output relationship are introduced [15]. As pointed out in [17], the practical consequence of the NFL theorems is that every demonstration that the generalization performance of one algorithm is better than another in a given suite of test data sets, is also an implied demonstration that it is worse on an alternative suite. In short, empirical success in supervised learning is always due to problem selection. So, even though it is tempting to interpret good generalization performance as a characteristic of the algorithm, in reality it only means that the algorithm was well matched to the suite of test data sets. In this sense, the DOSE framework can be seen as a principled approach to empirically investigate which combinations of data set characteristics are best matched (in a statistical sense) by specific algorithms.

Note that we do not observe an approximately balanced proportion of simulations in which one of the methods outperformed the other, as might seem to be implied by the NFL theorem. This is because, even though our simulations encompass a broad range of data set characteristics, they did not induce a uniform distribution over the space of target input-output relationships (i.e., the linear predictor in our case). For instance, the proportion of simulations where the linear predictor involved fewer features than the number of samples was highly skewed by our focus in the “large 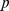, small 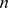
” setting. In other words, the very fact that we chose the parameter ranges for our simulations based on values observed in real genomic data sets imposes strong assumptions over the distribution of targets investigated in our simulations, skewing it away from a uniform distribution.

In the statistical analyses of the simulation results, we divided the ranges of the simulation parameters into ten equally spaced bins, and applied distance component analysis to test for differences in the response distributions of the groups, and permutation F-tests to detect the presence of 2-by-2 interactions between simulation parameters. Although our choice of 10 groups was arbitrary, we repeated our analyses using 5 and 15 groups as well (see Supplementary Tables in Text S5), and observed qualitatively similar results, suggesting that our analyses were robust to the number of bins used.

As pointed out in the Methods section, a simulation experiment represents a middle ground between computer and physical experiments. In particular, given the stochastic nature of the data generation process, replication might be a beneficial technique in the context of simulation experiments. However, because of limitations in time and computational resources, a question arises about whether is it better to adopt a single large design with, say, 10,000 input points or to run the simulations on 10 replicates of a smaller design with only 1,000 input points. In this work we choose to allocate our resources to a more detailed exploration of the experimental region by adopting a larger space filling design at the expense of performing replications.

The ability to clearly evaluate the relative merits of sophisticated statistical methods and machine learning algorithms in systematic and unbiased manner should be of broad utility to the computational biology community. In this paper, we proposed the use of DOSE as a general framework for the comparison of distinct algorithms used to solve a common machine learning problem and illustrate its application with the comparison of three widely used methods, whose predictive performance behavior is relatively well known. By showing that our simulations were able to provide new insights and recover expected behaviors, we demonstrate the value of adopting experimental design principles for the study of penalized regression models. A demonstration of the practical usefulness of DOSE in the comparison of a larger number of competing algorithms for regression (or classification) problems is, nonetheless, yet to be done.

## Methods

### Brief background on the methods under comparison

Ridge-regression [4] is a continuous shrinkage method that minimizes the residual sum of squares subject to a bound on the *L*_2_-norm of the coefficients. In ill defined problems, where the number of covariates, 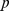, is larger than the sample size, 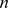, or where the covariates suffer from strong multi-collinearity, ridge-regression can be used to regularize the regression estimates, achieving better prediction accuracy through a bias-variance trade-off. Nonetheless, ridge-regression does not set any coefficients to 0 and does not produce an easily interpretable model.

The lasso [5] circumvents this problem by minimizing the residual sum of squares constrained to a bound on the *L*_1_-norm of the coefficients. The lasso penalty allows both continuous shrinkage and automatic variable selection to happen at the same time. However, it has been pointed out [6] that the lasso has three important limitations. First, when 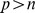, the lasso can select at most 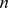 variables before it saturates. Second, if there is a group of covariates that are highly correlated the lasso tends to select a single variable. Third, when 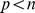 and the covariates are highly correlated, it has been shown empirically [5] that the predictive performance of the lasso is dominated by ridge-regression.

The elastic-net addresses these three problems by minimizing the residual sum of squares constrained by a penalty term that is a convex combination of the *L*_1_- and *L*_2_-norms, and thus is capable of performing automatic variable selection by setting some coefficients to 0, but can also select groups of correlated variables, and no longer suffers from the saturation issue that plagues the lasso.

### Computer experiments versus simulation experiments

Computer experiments [2], [3] are routinely used as a substitute for physical experiments when the latter are too expensive, unethical or infeasible. In computer experiments, a program is used to generate a response value associated with a set of input values. The program is deterministic, i.e., we obtain identical answers if we run the computer experiment twice using the same set of inputs. A consequence of this deterministic nature is that strategies employed in the design and analysis of computer experiments differ from the ones used in traditional physical experiments. For instance, well established practices in physical experiments such as replication, randomization and blocking are irrelevant in computer experiments. (In a computer experiment, a single observation at a given set of inputs gives us perfect information about the response at that particular set of inputs, hence replication is irrelevant. Furthermore, all experimental factors are known and randomization and blocking are not needed, since there are no uncontrolled variables that might affect the response in a systematic fashion, or might create uncontrolled heterogeneity.)

A simulation experiment represents a middle ground between computer and physical experiments. In simulation experiments, the inputs (simulation parameters) are used in the generation of the simulated data sets to which we apply and evaluate the performance of competing algorithms. The response variable is generally defined as a measure of relative performance of the competing methods, and our interest is to model the response as a function of the simulation parameters. Because the simulated data sets are generated from probability distributions, simulation experiments are not deterministic. Hence (and similarly to physical experiments) the use of replications might be helpful. On the other hand (and similarly to computer experiments) randomization and blocking are still irrelevant since all uncontrolled variables are represented by random noise associated with the generation process employed in the production of the simulated data sets, and cannot affect the response in any systematic fashion.

Similarly to both physical and computer experiments, where the scientific question to be answered determines the characteristics of the designed experiment, the types of statistical methods and algorithms to be compared dictate the specifics of the experimental design for a simulation study. Hence, the choice of simulation parameters (the inputs of the computer experiment) and the ranges of values investigated in the simulation study are case specific.

### Design of simulation experiments (DOSE)

#### Space filling design

In the context of design and analysis of computer experiments [2], an experimental design consists of a matrix of input values, with columns indexing the input types, and each row storing the input values for each simulation run. The region comprising the input values we want to study is called the experimental region. A multi-dimensional point in the experimental region corresponds to a specific set of input values. In situations were we don't know a priori the true relation between input values and the response variable under study, it is reasonable to adopt a design that provides information about all portions of the experimental region. Space filling designs are a popular choice in this context since they attempt to spread out the input values as evenly as possible across the entire experimental region. Furthermore, because of the deterministic nature of computer experiments, space filling designs use a single observation at any set of inputs so that each simulation is run with a unique combination of parameter values. (We point out, however, that in the context of a simulation experiment, it makes sense to have replications of the parameter value combinations, due to the stochastic nature of the simulated data.)

For our simulation study, we adopted a space filling design composed of 10,000 5-dimensional input points representing sample size, number of covariates, true model sparsity, signal-to-noise ratio, and correlation. Among the several available strategies to create a space filling design [2], we adopted a Latin hypercube design (LHD) optimized according to the maximin distance criterium [18], since it is relatively simple to draw samples from Latin hypercubes and there are robust and publicly available implementations of Latin hypercube samplers (namely, the **lhs** and **DiceDesign** R packages [19], [20]). Furthermore, LHD possess attractive properties under marginalization [2], making LHDs one of the most popular designs for computer experiments.

In a nutshell, a LHD in 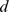 dimensions and 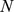 points is generated in the 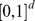 experimental region by dividing each one of its 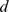 dimensions into 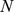 intervals, and selecting each one of the 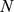 points in the experimental region such that, when projected onto any of the marginal dimensions, exactly one point is in each of the intervals for that dimension. In spite of this desirable marginalization property, a LHD will not necessarily represent a space filling design. Therefore, the usual practice in the design of computer experiments is to use a second criterium in order to select a LHD with good space filling properties [2]. Here, we adopt the maximin distance criterium which attempts to maximize the minimal distance between any two points in the design.

In the present study, we generated over 20 distinct design matrices using the maximin Latin hypercube sampler implemented in the **lhs** package (due to its computational efficiency and scalability), and selected the one with the best space filling properties measured according to the coverage and maximin distance metrics provided by the **DiceDesign** R package. The coverage criterion is defined as
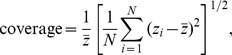
where 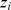 is the minimal distance between point 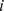 and the other points in the design, and measures the deviation of the design from a regular mesh (with smaller coverage values indicating more regular designs). The mindist criterium is defined as 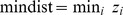, where 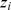 represents the minimal distance between the point 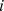 and the other points of the design. Higher mindist values correspond to more regular scaterring of design points. After we generated a space filling design on the 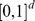 hypercube, we transformed the design points to the range of parameter values we are actually interested in studying.

#### A note on alternative designs involving categorical simulation parameters

The construction of the maximin Latin hypercube design adopted in this paper depends on a measure of distance between the input points. Therefore, it cannot be directly applied when some of the simulation parameters are categorical variables. In situations where all the simulation parameters are categorical we can use a full multifactorial experiment with crossed factors as an alternative to a space filling design. By adopting such a balanced full factorial design we ensure the orthogonality of the factors and protection against confounding of the simulation parameter effects on the relative performance response. The problem with such an approach is that the number of combinations of the crossed factors might be forbiddingly high even for a moderate number of simulation parameters and parameter levels. For instance, with 5 simulation parameters, and 10 levels per parameter, we would need to perform 10^5^ simulations in order to sample each one of the possible parameter level combinations. Hence, full factorials are only feasible when the number of levels per parameter is small. When this is not the case, a fractional factorial design might be used.

In situations where some of the parameters are categorical, while others are continuous, we have a few options. For instance, if we are willing to consider a factorial design with a small number of levels, we can divide the range of the continuous parameters into a small number of equally spaced discrete values, and treat the resulting ordinal variables as categorical factors in a full factorial design. For instance, if the range of a given continuous simulation parameter is [0, 3] we can divide this range into 3 equally spaced bins, 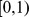, 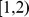, and 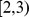, and use the midpoints 0.5, 1.5, and 2.5 as “categorical” parameter values in the simulation study. Alternatively, we can combine a space filling design generated from the continuous variables, with all possible level combinations of the categorical variables. For example, suppose we have two categorical inputs with two levels each, and three continuous parameters. We can generate 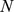 points from a space filling approach, using the three continuous parameters, in order to generate a 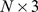 design matrix 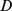, and then create a combined design, 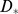, with 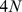 rows and 5 columns where each row of 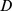, is combined with each one of the four combinations of the two categorical parameters levels in order to generate four rows in the design 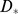.

#### Choice of simulation parameters and parameter ranges

An important step in the design of a simulation experiment is the choice of the relevant input simulation parameters, and the careful selection of the ranges of the simulation parameters to be investigated in the simulation study. Any characteristic of a data set that might impact the performance of a computational algorithm should be considered in the simulation study. For penalized regression models it is reasonable to expect that the sample size, the number of features, the sparsity of the true model generating the data, the amount of signal, the residual noise, and the amount of correlation between features, might affect the methods performance. For some of these parameters, it might be more natural to consider the ratio of two simulation parameters than each of the parameters on their own. For example, it is reasonable to expect the signal-to-noise ratio to capture the information provided by the signal and the noise parameters separately. In other cases, it is not completely clear whether the ratio would capture all the information provided by each of the parameters separately. For instance, even though we are frequently most interested in the number of features by sample size ratio, it is not clear whether the performance of the prediction algorithms would be the same in a data set containing 300 features and 100 samples, as in a data set containing 3,000 features and 1,000 samples. In these situations it is often advisable to run a pilot simulation study to investigate the issue. In the Supplement (Text S2) we present such a study, showing that, in fact, we can safely replace the signal and noise parameters by their ratios, whereas we should keep the number of features and sample size parameters separate.

Once we decide which parameters should be used as inputs in the simulation study, we need to carefully determine the experimental region to be explored in the study. Our approach was to select the parameter ranges to be as realistic and consistent as possible with observed ranges in real genomic data applications. For each one of the five inputs in our simulation study, we selected the parameter value ranges as follows:

1. Sample size, *n*, was selected in the discrete range 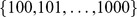. Note that sample sizes in genomic studies in the “large *p*, small *n*” setting, using high throughput “omics” data sets, typically comprise a few hundred samples, with few studies having more than 1,000 samples.
2. Number of features, *p*, was selected in the discrete range 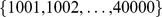. The upper limit on this range encompasses the number of probes generally sampled in RNA microarrays. Even though some technologies, such as SNP arrays, can produce much larger number of genomic features, it is often the case that some filtering is applied to screen out non-informative features, so that the selection of 40,000 as an upper bound for the range of *p*, is still a realistic choice.
3. Saturation parameter, 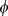, that specifies the probability that a covariate enters the regression model, was selected in the continuous interval 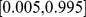. This choice ensures we are able to simulate from highly sparse to highly saturated models.
4. Signal-to-noise parameter, 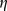, was selected in the continuous range 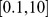, with one half of the simulations in the 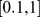 interval addressing the cases where the noise was higher than the signal, and the other half in the interval [1], [10] addressing the cases where signal was higher than the noise. This range cover cases where the signal is 10 times lower to 10 times higher than the noise.
5. Feature correlation parameter, 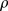, was selected in the continuous range 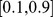. This parameter controls the amount of correlation between feature blocks according to a Toeplitz structure (see next section for further details). This range covers the full spectrum from weak to strong correlation structures.

As described previously, we first generated a maximin LHD in the 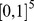 hypercube, and then mapped the values in the 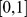 intervals back to the range of each of the simulation parameters we are interested to study. For 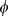 and 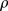 we adopted a simple linear transformation
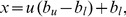
(1)where 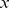 represents the transformed parameter value, 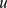 represents the original value in the 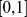 scale, and 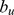 and 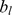 represent, respectively, the upper and lower bounds of the parameter range of interest. For 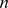 and *p*, we adopt the same linear transformation with the additional step of rounding the transformed parameter value to an integer. For 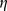 we apply the following transformation
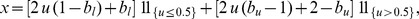
where 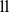 represents the indicator function. Observe that this transformation maps 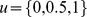 into 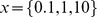. The transformed simulation parameter values are then combined into the experimental design matrix, *D*, with columns indexing the simulation parameters, and rows indexing the input sets for each simulation.

#### Stochastic data generation process

After we generated the experimental design, *D*, as explained in the previous sections, we generated the response and covariates data sets, for each simulation, as follows (see Figure 1 for a graphical model representation of our data generation process):

1. Given the values of *n*, *p*, and 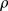, in a particular row of the design *D*, we simulate the covariate data matrix, 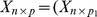, 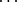, 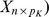, as *K* separate matrices, 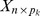, generated independently from 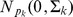 distributions, where the covariance matrix was generated according to a Toeplitz structure with 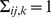, for 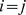, and 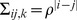, for 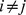. The number of covariates, 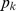, in each of these matrices were randomly chosen between 20 and 300 under the constraint that 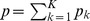.
2. Given the values of 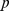, 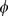, and 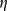, we computed each regression coefficient, 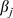, 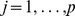, as 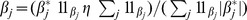, where 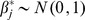, and 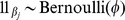. Note that, by defining 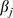 as above, we guarantee that the signal-to-noise ratio (defined as the average signal, 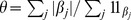, divided by the residual noise, set to 1, in this case), is equal to the sampled value of 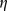, and that, on average, 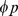 regression coefficients will be non-zero.
3. Finally, given the computed covariates matrix and regression coefficients vector, we computed the independent variable vector, 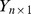, as 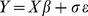, where 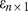 is a vector of standard normal error variables, and 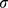 is set to 1.

Each simulated data set was composed of the independent variable vector, and the matrix of covariates. Each data set was split in two parts, generating independent training and testing data sets (we actually simulated data-sets of size 2*n*, so that the training and testing data sets had *n* samples, ranging from 100 to 1000). Both response and covariates were centered and scaled. Finally, we would like to point out that we generate the covariates data using the blocked correlation structure described in item 1 above for two reasons: (i) blocked structures are often seen in real data sets, where variables are usually grouped in blocks with different sizes; and (ii) it is computationally more efficient to simulate the covariate data as blocks of correlated variables, since data generation from a multivariate normal distribution involves performing a Choleski decomposition of the associated covariance matrix, a computationally challenging task for large matrices (recall that covariance matrices dimensions would range from 1,001 to 40,000 in our simulations). At this point, a natural question is whether the blocking structure in addition to the strength of the correlation (as measured by the simulation parameter 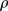) can affect predictive performance of the methods under study. To address this question we performed a pilot simulation study (see Text S3 in the Supplement for details). We found out that blocking has a minor effect on the predictive performance of ridge-regression, lasso, and elastic-net, but no effect in their relative performance, as measured by differences in MSE scores. In view of these findings, we decided to leave the blocking structure as an uncontrolled variable, and randomly select the block sizes (as described in item 1 above) in a range where the Choleski decomposition can be performed efficiently.

### Model fitting and performance evaluation details

The lasso and elastic-net fits were performed with the **glmnet** R package [21]. Following the parametrization of the elastic-net penalty in [21], 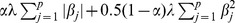, we adopted the default 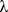 grid of 100 values generated automatically by the **cv.glmnet** function (for both the lasso and elastic-net), and an 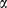 grid given by the sequence 0.001, 0.01, 0.10, 0.15, 0.20,…, 0.95, for the elastic-net. (For the lasso we set 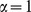.) The ridge regression model fit was performed with a modified version of the **ridge.lm** function from the **MASS** R package [22], and the determination of the tuning parameter grid for its penalty is described in Text S4 on the Supplement. For all methods we optimized the tuning parameters in the training data set using 10-fold cross-validation, and evaluated their predictive ability in the testing data set, using the MSE, 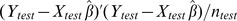, where 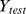 and 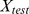 represent the response and covariate testing data, and 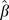 is estimated from the training data. Because we scale the response variables, the predictions from an intercept-only model (which contains no features and, therefore, completely ignores the information contained in the input variables) will generate a MSE score close to 1.

### Data analysis

In order to understand how the simulation parameters affect the absolute performance of a given method 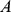 we adopted 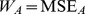 as the response (output variable), and the simulation parameters as the input variables. For the relative performance comparison of any two distinct methods, we again used the simulation parameters as the input variables but defined the response, 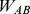, as 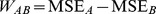. In both cases we assume that the response is affected by an unknown error term, 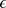, such that E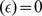 and Var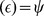, and is related to the covariates (i.e, our simulation parameters) by a function, 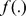, according to 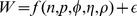. For the examples presented in the Results section we observed that the simulation parameters and the response values were related in a non-linear fashion (see Figures 3, 5, 7, 9, 11, and 13). Furthermore, the distribution of our response variables is not Gaussian (Figure S2 in the Supplement). Therefore, assuming a linear model with Gaussian errors is inadequate.

In order to account for non-linearity and lack of normality, we discretize the ranges of simulation parameters into a number of equally spaced groups, and assess the statistical significance of trends observed in the data using distance components analysis (DISCO) [10]. While standard one-way ANOVA tests the null hypothesis that the group means are zero, DISCO tests the more general null that the groups distributions are equal, 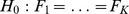, versus the composite alternative 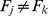 for some 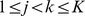, where 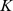 represents the number of groups and 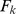 the distribution function of the *k*th group. While ANOVA is only able to detect shifts in mean, DISCO can detect changes in the dispersion of the groups as well. The DISCO test statistic is a function of powered Euclidean norms between all pairs of sample elements, for any power 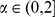. In the special case of 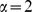 the DISCO test statistic reduces to the usual ANOVA F-statistic (which measures dispersion by considering squared distances of sample elements from the sample mean). P-values for the DISCO null hypothesis are computed via a permutation test, and hence is a non-parametric test. DISCO is implemented in the **energy** R package [25], and we adopted 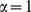 in our analyzes.

Except for the signal-to-noise parameter, Figures 3, 5, 7, 9, 11, and 13 show a fair amount of variation in the shapes of the boxplots across the binned parameter ranges. This heterogeneity in the distributional form of the response variable suggests the presence of parameter interactions [23]. Although DISCO analysis readily generalizes to the analysis of multifactorial-ANOVA with interactions [10], the current R implementation only handles one-way models. In order to test for the presence of 2-by-2 interactions we adopted permutation tests based on the two-way ANOVA interaction F-statistic to check whether the curves in an interaction plot are parallel.

Following reference [24], the permutation p-values for our non-parametric interaction tests (and for DISCO as well) are computed as
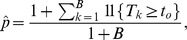
where 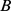 represents the number of permutations, 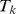 represents the test statistic computed at permutation 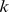, and 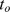 represents the observed test statistic. Observe that the assumption of exchangeability of observations under the null (required by the permutation tests) seems reasonable in our context.

### Data and code availability

Given the scale of our study, the simulations were performed with cluster computing in the AWS cloud using the **rredis** [26] and **doRedis** [27] R packages. The predictive performance data used in the paper, that is, the MSE values derived from the 10,000 simulations, is available in Synapse under the project DOSE (https://www.synapse.org/#!Synapse:syn2177826). All the open-source code to simulate the data sets, fit the penalized regression models, and analyze the predictive performance results is deposited in Github and can be accessed from the DOSE project in Synapse.

## Supporting Information

Figure S1
**Performance of ridge-regression, lasso, and elastic-net under sparse and saturated models.** Scatter plots of the MSE*_R_* versus MSE*_L_* (panel a) and MSE*_R_* versus MSE*_E_* (panel b) colored according to the true model sparsity. Blue dots show the simulations where the number of covariates entering the true model was equal or smaller than the sample size. In total, only 476 (out of the 10,000) simulations showed this pattern. Red dots show the reverse situation (9,524 simulations). Note that in both panels a and b, the red dots tend to be concentrated above (but close) to the diagonal line, whereas the blue dots tend to be located below but dispersed away from the diagonal. This suggests that for sparse models lasso and elastic net tend to perform better than ridge, and often times considerably better (note the concentration of points close to 0 in the y-axis, but ranging from 0 to 1 in the x-axis). For more saturated models, on the other hand, ridge regression tends to be better, but the difference in performance is not accentuated (especially for the elastic net - panel b). Panel c shows the distribution of the actual number of covariates that entered the true model across the 10,000 simulations. At each simulation, the actual number of covariates was sampled from a binomial distribution with 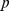 trials and probability of success given by 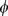.(EPS)

Figure S2
**Response distributions.** Panels a, c, and e show, respectively, the response distributions of 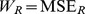 (ridge-regression), 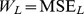 (lasso), and 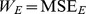 (elastic-net), used in the absolute performance analyses. Panels b, d, and f show, respectively, the response distributions 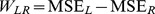 (lasso vs ridge), 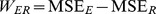 (elastic-net vs ridge), and 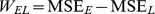 (elastic-net vs lasso), used in the relative performance analyses. In all cases the responses do not follow a normal distribution.(EPS)

Text S1
**Additional investigations on the** 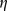 **parameter.** Additional simulation experiments investigating the effect of the signal-to-noise parameter under four distinct ranges of 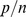 ratios.(PDF)

Text S2 **Pilot studies: original parameters versus parameter ratios.** Pilot simulation study investigating whether the signal-to-noise and number-of-features-to-sample-size ratio parameters could be used in place of the respective original parameters.
(PDF)

Text S3 **Pilot studies: the effect of blocked correlation structure.** Pilot simulation study investigating whether blocking structure, in addition to correlation strength, can affect predictive performance of the methods under study.
(PDF)

Text S4 **Tuning parameter grid for the ridge-regression.** Description of the automatic/data-driven approach used to determine the tuning parameter grid for ridge-regression.
(PDF)

Text S5 **Supplementary tables.** Permutation tests for equality of the group distributions using distance components analysis, and permutation F-tests for the presence of 2-by-2 interactions, using 5, 10, and 15 bins. Table S1 compares ridge-regression vs lasso. Table S2 compares ridge-regression vs elastic-net. Table S3 compares lasso vs elastic-net.
(PDF)
