# Supplementary material for: Simulation Studies as Designed Experiments: The Comparison of Penalized Regression Models in the “Large p, Small n” Setting
Source: PLoS One. 2014 Oct 7;9(10):e107957. doi: 10.1371/journal.pone.0107957 (PMC4188526; doi:10.1371/journal.pone.0107957)
Supplement: Text S1 — Additional investigations on the parameter. Additional simulation experiments investigating the effect of the signal-to-noise parameter under four distinct ranges of ratios. (PDF) [file pone.0107957.s003.pdf]

Text S1. Additional investigations on the η parameter

Figures 3d, 5d, and 7d in the main text show that the signal-to-noise parameter have a weak (although statistically significant) effect on the predictive performance of ridge-regression, lasso, and elastic-net. Figure 1 presents results from an additional set of simulations where we investigated the effect of the signal-to-noise parameter (η) under four distinct ranges of p/n ratios. (A detailed description of these additional simulation studies is provided in the caption of the figure.)

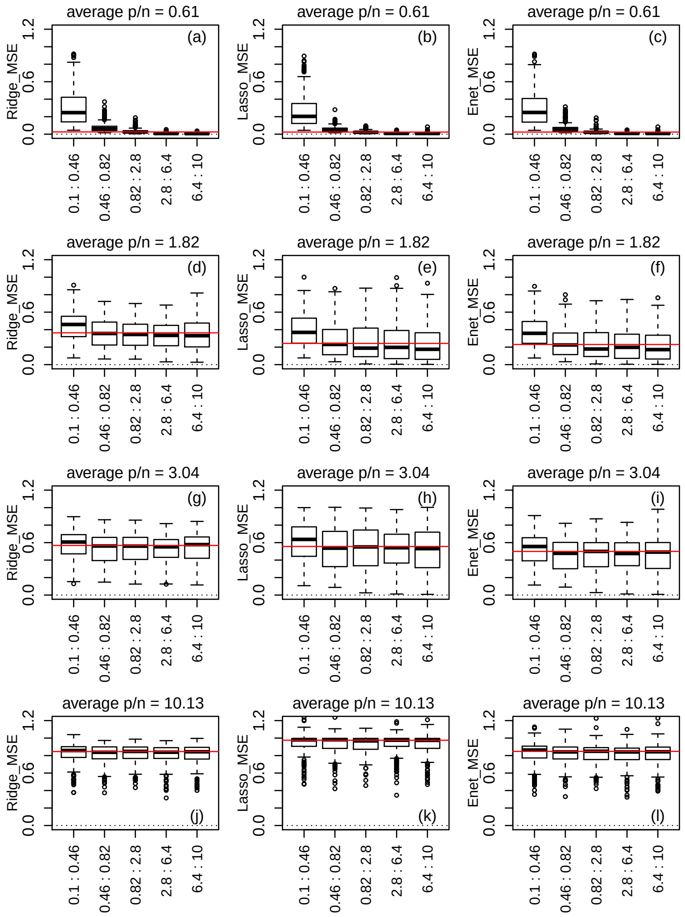

Figure 1. Distributions of the MSE responses for ridge, lasso and elastic-net as a function of η. The results were derived from 4 distinct DOSE experiments. Each experiment was based on 1,000 syntectic data sets generated according to the stochastic data generation process described in the main text with: sample size, n, selected in the discrete range $\{200, \ldots, 300\}$; saturation parameter, ϕ, selected in the continuous interval $[0.1, 0.9]$; signal-to-noise parameter, η, in the continuous range $[0.1, 10]$; and feature correlation parameter, ρ, in the continuous range $[0.1, 0.9]$. For the first experiment (panels a-c) the number of features, p, was selected in the discrete range $\{100, \ldots, 200\}$. For the second experiment (panels d-f) p was selected in the discrete range $\{300, \ldots, 600\}$. For the third experiment (panels g-i) p was selected in the discrete range $\{600, \ldots, 900\}$. Finally, for the fourth experiment (panels j-l) p was selected in the discrete range $\{2000, \ldots, 3000\}$. The red horizontal line represents the median of the response distribution. The dotted line is set at 0.

Figure 2 presents the distributions of the p/n ratios for the four additional simulation studies (panels a-d), as well as the p/n ratio distribution of the main simulation study presented in the paper.

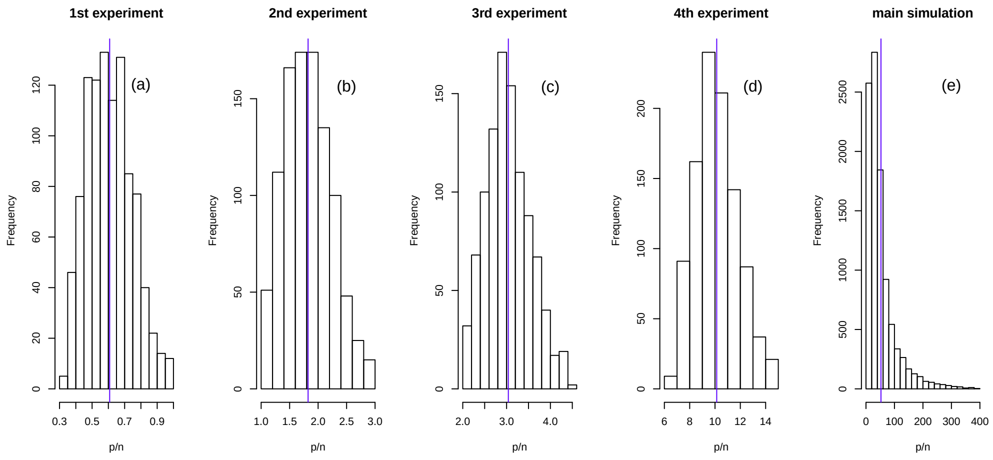

Figure 2. Distributions of the p/n ratios for the 4 additional experiments (panels a-d), as well as for the main text simulation. The vertical blue lines represent the respective average of p/n ratios.

Figure 1 reports the distributions of the MSE responses for ridge, lasso and elastic-net as a function of the η parameter. Panels a-c present the results for the first experiment where $p \leq n$ (average $p/n = 0.61$). In this well conditioned situation we observe a strong influence of the η parameter on the predictive performance of the methods, with performance improvement increasing as a function of the amount of signal in the data (as one would expect). However, this strong influence quickly decreases as we increase the p/n ratio (see panels d-f where the average $p/n = 1.82$; and panels g-i with average $p/n = 3.04$), and is practically undetectable by eye for larger p/n ratios (see panels j-l where the average $p/n = 10.13$).

These results suggest that the lack of strong influence of the η parameter, observed in our main simulations, is due to the overwhelming effect of the p parameter. Figure 2e shows that the average p/n ratio in our simulations is 52.58 (blue line), and that quite a few simulations achieve p/n ratios larger than 100 (and some reaching almost 400).
