# Supplementary material for: Simulation Studies as Designed Experiments: The Comparison of Penalized Regression Models in the “Large p, Small n” Setting
Source: PLoS One. 2014 Oct 7;9(10):e107957. doi: 10.1371/journal.pone.0107957 (PMC4188526; doi:10.1371/journal.pone.0107957)
Supplement: Text S2 — Pilot studies: original parameters versus parameter ratios. Pilot simulation study investigating whether the signal-to-noise and number-of-features-to-sample-size ratio parameters could be used in place of the respective original parameters. (PDF) [file pone.0107957.s004.pdf]

Text S2. Pilot studies: original parameters vs parameter ratios

An important step in the design of a simulation experiment is the choice of the input simulation parameters. In some situations, the ratio of two simulation parameters might represent a more fundamental input than each of the parameters on their own. For instance, it is reasonable to expect the signal-to-noise ratio to capture the information provided by the signal, θ, and the noise, σ, parameters separately. In this case, we can simply set one of the simulation parameters to a fixed value and vary the other one. Reducing the number of simulation parameters is always advantageous, since: (i) it is easier to generate an optimized Latin hypercube design of lower dimension, when adopting a space filling design; and (ii) we can considerably reduce the number of simulations, when running a full factorial experiment.

However, for some parameters, it is not clear whether one should replace the original parameters by their ratio. For example, in the case of the number of features, p, and sample size, n, it is not clear whether the p/n ratio would be a better input choice, since it is hard to tell whether the performance of the prediction algorithms would be the same in a data set containing 300 features and 100 samples, as in a data set containing 3,000 features and 1,000 samples.

In order to investigate this question we performed a pilot simulation study evaluating the predictive performance of the ridge-regression, lasso, and elastic-net algorithms in five distinct simulation settings, $(p, n) = \{(300, 100), (600, 200), (900, 300), (1200, 400), (1500, 500)\}$, where, nonetheless, the p/n ratio was constant and equal to 3. For each one of these five settings, we simulated 500 data sets, generating a total of 2,500 simulations. The other simulation parameters were fixed as $\eta = 1$, and $\phi = \rho = 0.5$ (see the Methods section in the main text for a description of the simulation parameters and of the data generation process). Figure 1 present the results.

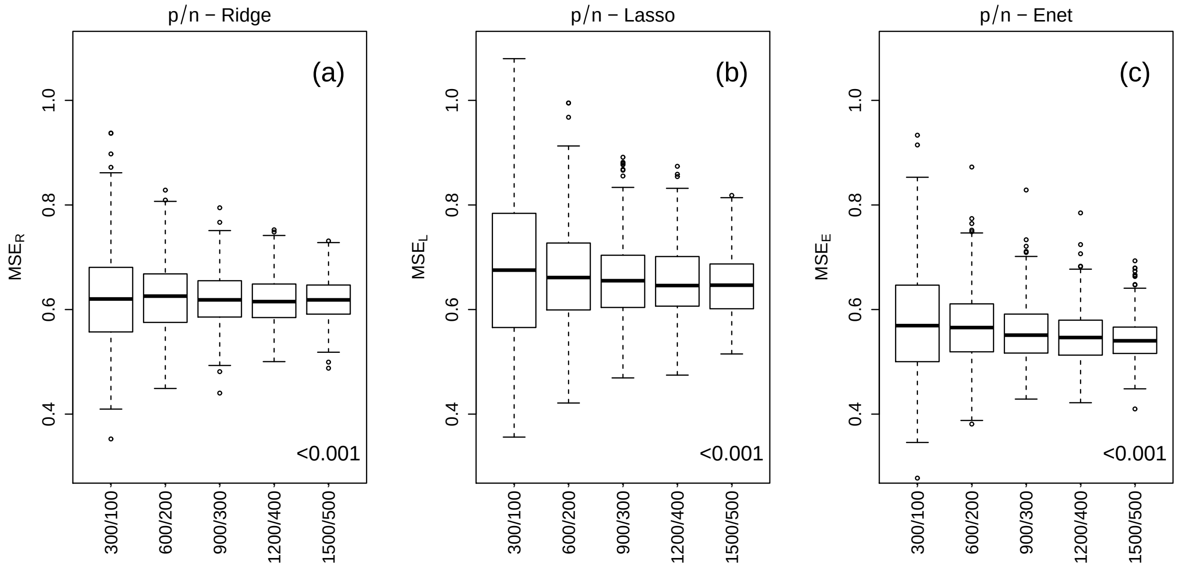

Figure 1. Pilot study evaluating whether p and n or the p/n ratio should be adopted as input for the simulation study. Panels a-c present boxplots of the MSE distributions under five distinct simulation settings for the ridge-regression, lasso, and elastic-net methods, respectively. For each simulation setting we generated 500 data sets using $\eta = 1$, $\phi = \rho = 0.5$, but varying p and n according to $(p, n) = \{(300, 100), (600, 200), (900, 300), (1200, 400), (1500, 500)\}$. Note that $p/n = 3$ in all simulation settings. Permutation p-values for the DISCO null hypothesis that all MSE distributions are the same are shown in the lower right corners of the panels.

For all methods, Figure 1 clearly shows distinct distributions for the predictive performance scores (MSE) for different choices of p and n, corresponding to the same p/n ratio. Note the different boxplot spreads for all methods, and the different median values for the lasso and elastic-net algorithms (panels b and c). In order to formally test the null hypothesis that the MSE distributions are the same across all five simulation settings we employed the distance components (DISCO) permutation test [1]. DISCO represents a non-parametric generalization of the ANOVA, that tests the more general null hypothesis that the group distributions, and not only their means are the same. The test was performed using the `disco` function (setting the exponent on the Euclidian distance parameter to 1) of the `energy` R package. For all three methods we rejected the null hypothesis (DISCO permutation p-values < 0.001). This pilot study suggests we should incorporate the p and n parameters as separate inputs in the simulation study.

In order to confirm our expectation that the signal-to-noise ratio could be used alone to replace the signal and noise parameters as inputs in the simulation experiment, we performed a similar pilot study. Again we considered five distinct simulation settings, $(\theta, \sigma) = \{(1,1), (3,3), (5,5), (7,7), (9,9)\}$, where, nonetheless, the $\eta = \theta/\sigma$ ratio was constant and equal to 1. Again, for each one of these five settings, we simulated 500 data sets, generating a total of 2,500 simulations. The other simulation parameters were fixed as $n = 300$, $p = 900$, and $\phi = \rho = 0.5$.

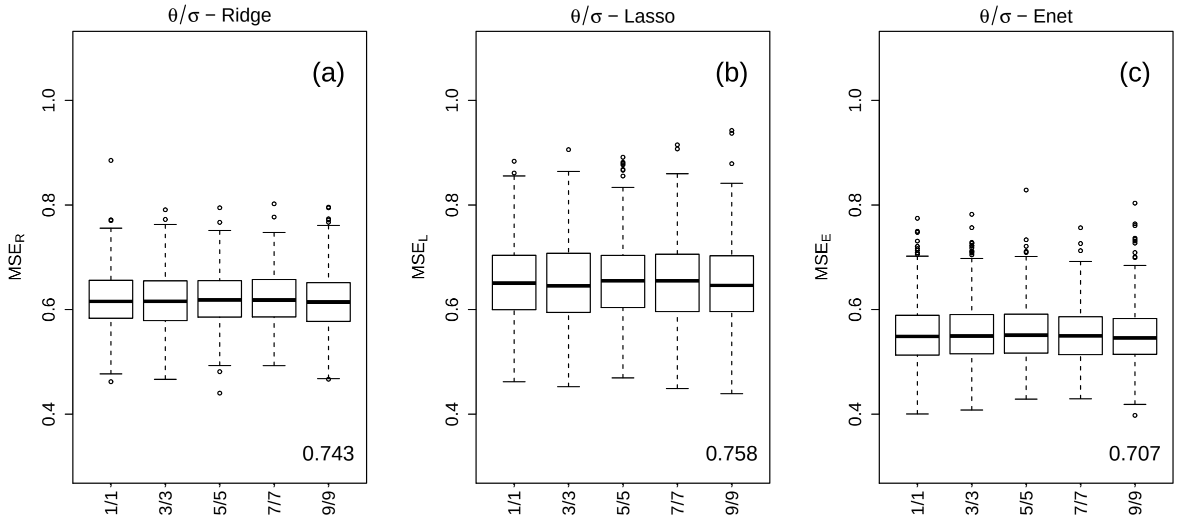

Figure 2. Pilot study evaluating whether θ and σ or the signal-to-noise ratio should be adopted as input for the simulation study. Panels a-c present boxplots of the MSE distributions under five distinct simulation settings for the ridge-regression, lasso, and elastic-net methods, respectively. For each simulation setting we generated 500 data sets using $n = 300$, $p = 900$, $\phi = \rho = 0.5$, but varying η and σ according to $(\theta, \sigma) = \{(1,1), (3,3), (5,5), (7,7), (9,9)\}$. Note that $\theta/\sigma = 1$ in all simulation settings. Permutation p-values for the DISCO null hypothesis that all MSE distributions are the same are shown in the lower right corners of the panels.

Figure 2 presents the results. For all methods, we clearly see that the MSE distributions are quite close across all five settings. The DISCO analysis corroborates this finding with non-significant p-values for all methods.

In order to further investigate whether this finding is characteristic to the particular choice of simulation parameter values, or if it would hold in general, for other choices of values, we ran an additional pilot study employing a space filling design (a Latin hypercube, optimized according to the maximin distance

criterium - see Methods section in the main text for details) and evaluating the relative performance of the three methods as the response variable. In this additional pilot study we evaluated the same five average signal and residual noise settings as before, $(\theta, \sigma) = \{(1,1), (3,3), (5,5), (7,7), (9,9)\}$, but adopted the following ranges for the other simulation parameters: $n = \{100, 101, \ldots, 500\}$, $p = \{501, 502, \ldots, 1000\}$, and $\phi = \rho = [0.1, 0.9]$.

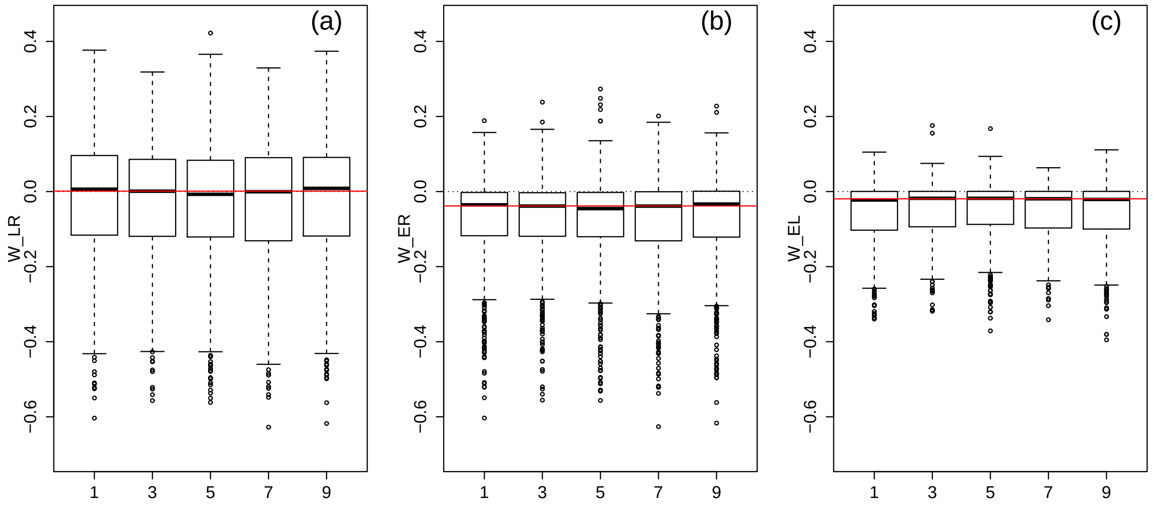

Figure 3. Pilot study evaluating the effect of the average signal and residual noise on the relative predictive performance of ridge-regression, lasso, and elastic-net. Panels a-c present, respectively, the boxplots of the pairwise comparisons of lasso vs ridge-regression, elastic-net vs ridge-regression, and elastic-net vs lasso. The relative performance responses in the y-axes were defined as $W_{AB} = MSE_A - MSE_B$. The horizontal red line shows the overall median, while the dotted line is set at zero.

Figure 3 shows the relative performances (as measured by differences in MSE scores) for the three pairwise comparisons of the three methods. Panels a to c show no significant distribution differences across all five simulation settings (DISCO p-values were, respectively, 0.989, 0.993, and 0.883). These pilot studies confirm our expectation that the θ and σ parameters could be replaced by the more fundamental $\eta = \theta/\sigma$ ratio parameter as input in the simulation study.

References

1. Rizzo ML, Szekely GJ (2010) Disco analysis: a nonparametric extension of analysis of variance. Annals of Applied Statistics 4: 1034-1055.
