# Supplementary material for: Simulation Studies as Designed Experiments: The Comparison of Penalized Regression Models in the “Large p, Small n” Setting
Source: PLoS One. 2014 Oct 7;9(10):e107957. doi: 10.1371/journal.pone.0107957 (PMC4188526; doi:10.1371/journal.pone.0107957)
Supplement: Text S3 — Pilot studies: the effect of blocked correlation structure. Pilot simulation study investigating whether blocking structure, in addition to correlation strength, can affect predictive performance of the methods under study. (PDF) [file pone.0107957.s005.pdf]

# Text S3. Pilot studies: the effect of blocked correlation structure

In the generation of the simulated data, we simulate the covariate data matrix, $X_{n \times p} = (X_{n \times p_1}, \ldots, X_{n \times p_K})$, as $K$ separate matrices, $X_{n \times p_k}$, generated independently from $\text{N}_{p_k}(0\,, \Sigma_k)$ distributions, where the covariance matrix was generated according to a Toeplitz structure with $\Sigma_{ij,k} = 1$, for $i = j$, and $\Sigma_{ij,k} = \rho^{|i-j|}$, for $i \neq j$. The number of covariates, $p_k$, in each of these matrices were randomly chosen between 20 and 300 under the constraint that $p = \sum_{k=1}^{K} p_k$.

We adopt this blocked correlation structure for two reasons: (i) blocked structures are often seem in real data sets, where variables are usually grouped in blocks with different sizes; and (ii) it is computationally more efficient to simulate the covariate data as blocks of correlated variables, since generating data from a multivariate normal distribution involves performing a Choleski decomposition of the associated covariance matrix, a computationally challenging task for large matrices. At this point, a natural question is whether the blocking structure in addition to the strength of the correlation (as measured by the simulation parameter $\rho$) can affect predictive performance of the methods under study.

To address this question we performed a pilot simulation study evaluating the predictive performance of the ridge-regression, lasso, and elastic-net algorithms in five distinct block size settings, namely, {50, 100, 150, 200, 300}. For all settings we fixed the number of features to 600 so that in the first set the covariate data is generated using 12 blocks of size 50, while in the last setting the covariate data is generated using 2 blocks of size 300. For each one of these settings, we simulated 500 data sets, generating a total of 2,500 simulations. The simulation parameters were fixed as $n = 300$, $p = 600$, $\phi = 0.5$, $\eta = 1$, and $\rho = 0.9$ (see the Methods section in the main text for a description of the simulation parameters and of the data generation process). Figure 1 present the results.

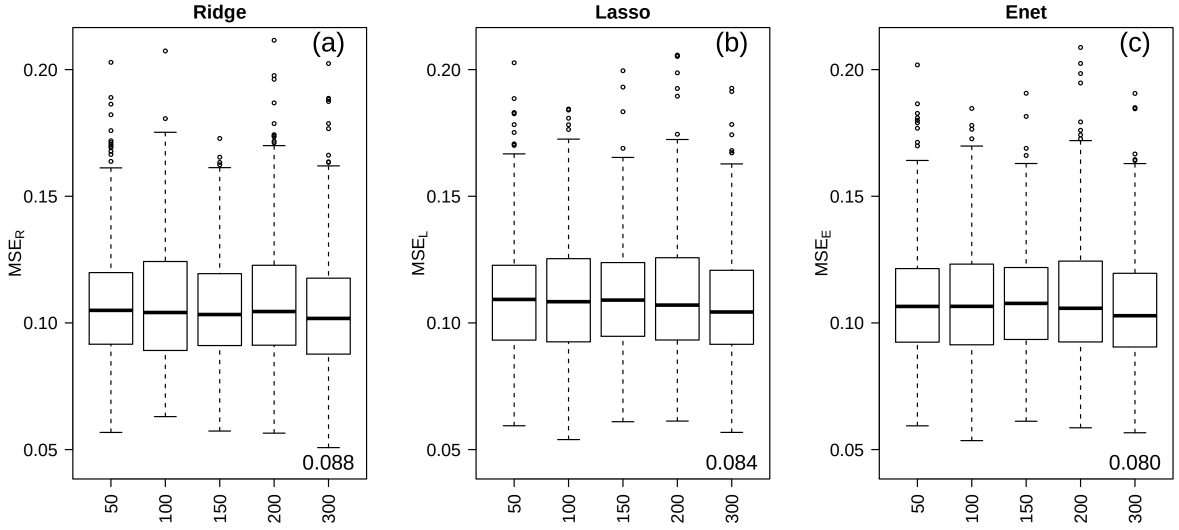


**Figure 1. Pilot study evaluating the effect of block size on predictive performance.** Panels a-c present boxplots of the of the MSE distributions under five distinct block sizes for the ridge-regression, lasso, and elastic-net methods, respectively. For each simulation setting we generated 500 data sets using $n = 300$, $p = 600$, $\phi = 0.5$, $\eta = 1$, $\rho = 0.9$, but varying the block sizes according to the values shown in the x-axis. Permutation p-values for the DISCO null hypothesis that all MSE distributions are the same are shown in the lower right corners of the panels.

For all methods Figure 1 shows marginally significant effects of block size on predictive performance

according to the DISCO analysis [1]. In order to further investigate whether this weak effect was characteristic to the particular choice of simulation parameter values, or if it would have an average stronger effect for other choices of values, and more importantly, whether the relative performance of the methods would be affected by block size, we ran an additional pilot study evaluating the same five block sizes, in data sets composed of 600 features but with sample sizes varying in the range $\{100, 101, \ldots, 300\}$, sparsity and feature correlation parameters varying in the range $[0.1, 0.9]$, and signal-to-noise varying in the range $[0.1, 10]$. A space filling design based on a Latin hypercube, optimized according to the maximin distance criterium, was employed in this pilot (see the Methods section in the main text for details on this experimental design). Figure 2 presents the results.

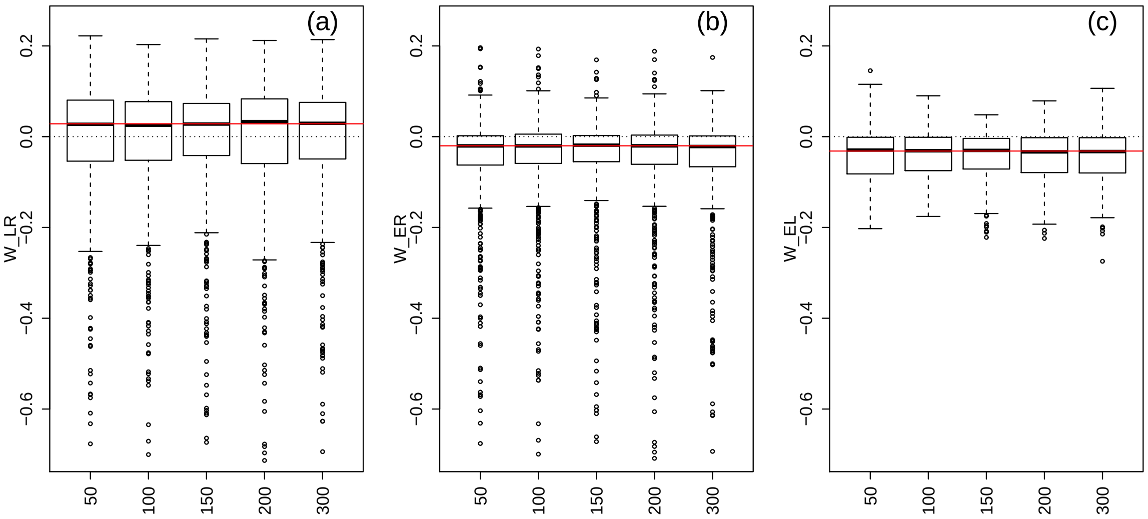


**Figure 2. Pilot study evaluating the effect of block size on the relative predictive performance of ridge-regression, lasso, and elastic-net.** Panels a-c present, respectively, the boxplots of the pairwise comparisons of lasso vs ridge-regression, elastic-net vs ridge-regression, and elastic-net vs lasso. The relative performance responses in the y-axes were defined as $W_{AB} = MSE_A - MSE_B$. The horizontal red line shows the overall median, while the dotted line is set at zero.

Figure 2 shows the relative performances (as measured by differences in MSE scores) for the three pairwise comparisons of the three methods. Panels a to c clearly show that block size has no significant effect on the relative performance of the methods (DISCO p-values were, respectively, 0.951, 0.999, and 0.971). These findings suggest to we can leave the blocking structure as an uncontrolled variable, and randomly select the block sizes in a range where the Choleski decomposition can be performed efficiently.

# References

1. Rizzo ML, Szekely GJ (2010) Disco analysis: a nonparametric extension of analysis of variance. Annals of Applied Statistics 4: 1034-1055.
