# Supplementary material for: Simulation Studies as Designed Experiments: The Comparison of Penalized Regression Models in the “Large p, Small n” Setting
Source: PLoS One. 2014 Oct 7;9(10):e107957. doi: 10.1371/journal.pone.0107957 (PMC4188526; doi:10.1371/journal.pone.0107957)
Supplement: Text S4 — Tuning parameter grid for the ridge-regression. Description of the automatic/data-driven approach used to determine the tuning parameter grid for ridge-regression. (PDF) [file pone.0107957.s006.pdf]

Text S4. Tuning parameter grid for ridge-regression

For the sake of completeness, in this section we describe the rationale behind the automatic/data-driven determination of the tuning parameter grid in our implementation of the ridge-regression (originally proposed in [1]). It is a simple adaptation of the approach adopted in the `glmnet` package for the default determination of the λ grid in the lasso and elastic-net algorithms. Following reference [2], the basic idea is to: (i) determine λ_{max}, as the λ value such that the largest regression coefficient is equal in absolute value to a certain small constant κ; (ii) determine the smallest λ value in the grid as $\lambda_{\text{min}} = \epsilon\,\lambda_{\text{max}}$, where ϵ is another small constant; and (iii) determine the λ grid as a sequence of K values of λ decreasing from λ_{max} to λ_{min} on the log scale. Explicitly, we set the lambda grid as follows: (a) create a decreasing sequence of K equally spaced values in the interval $\big[\log(\lambda_{\text{max}}), \log(\lambda_{\text{min}})\big]$; and (b) take the exponential of each of value in the sequence.

Next, we describe the derivation of λ_{max}. Considering the singular value decomposition of $X = UDV'$, we can re-express the ridge estimator as

$$\hat{\beta} = (X'X + \lambda I)^{-1} X'y = V(D^2 + \lambda I)^{-1} D U' y$$

or

$$\hat{\beta}_j = \frac{d_j}{d_j^2 + \lambda} V_j\, U'\, y \ ,$$

for $j = 1, \ldots, n$ (and zero for $j = n+1, \ldots, p$) where V_j represents the jth row of V. Our goal is to find λ such that $\max_j(|\hat{\beta}_j|) = \kappa$. Since

$$|\hat{\beta}_j| = \frac{d_j}{d_j^2 + \lambda} |V_j\, U'\, y| \ \leq \ \frac{d_j}{\lambda} |V_j\, U'\, y|$$

for all d_j, it follows that

$$\kappa = \max_j \left(\frac{d_j}{d_j^2 + \lambda} |V_j\, U'\, y| \right) \ \leq \ \frac{1}{\lambda} \max_j \left(d_j |V_j\, U'\, y| \right)$$

so that

$$\lambda \ \leq \ \frac{1}{\kappa} \max_j \left(d_j |V_j\, U'\, y| \right)$$

and we take $\lambda_{\text{max}} = \max_j \left(d_j |V_j\, U'\, y| \right) / \kappa$. In our simulations we adopted $\kappa = 10^{-3}$, $\epsilon = 10^{-6}$, and $K = 100$.

References

1. Chaibub Neto E, Jang IS, Friend SH, Margolin AA (2014) The stream algorithm: computationally efficient ridge-regression via Bayesian model averaging, and applications to pharmacogenomic prediction of cancer cell line sensitivity. Pacific Symposium on Biocomputing 19: 27-38.

2. Friedman J, Hastie T, Tibshirani R (2010) Regularization paths for generalized linear models via coordinate descent. Journal of Statistical Software 33: 1-22.
