# Supplementary material for: Simulation Studies as Designed Experiments: The Comparison of Penalized Regression Models in the “Large p, Small n” Setting
Source: PLoS One. 2014 Oct 7;9(10):e107957. doi: 10.1371/journal.pone.0107957 (PMC4188526; doi:10.1371/journal.pone.0107957)
Supplement: Text S5 — Supplementary tables. Permutation tests for equality of the group distributions using distance components analysis, and permutation F-tests for the presence of 2-by-2 interactions, using 5, 10, and 15 bins. Table S1 compares ridge-regression vs lasso. Table S2 compares ridge-regression vs elastic-net. Table S3 compares lasso vs elastic-net. (PDF) [file pone.0107957.s007.pdf]

Tables S1. Supplementary tables

	5 bins		10 bins		15 bins	
par.	obs. stat.	p-value	obs. stat.	p-value	obs. stat.	p-value
n	58.297	<0.001	28.003	<0.001	18.329	<0.001
p	194.965	<0.001	99.312	<0.001	69.788	<0.001
ϕ	115.574	<0.001	78.062	<0.001	58.982	<0.001
η	0.942	0.485	0.949	0.541	0.956	0.585
ρ	183.323	<0.001	85.745	<0.001	56.313	<0.001
$n : p$	9.543	<0.001	3.948	<0.001	2.199	<0.001
$n : \phi$	22.016	<0.001	8.321	<0.001	4.621	<0.001
$n : \eta$	0.698	0.808	1.002	0.482	0.906	0.842
$n : \rho$	1.753	0.030	0.996	0.490	0.974	0.600
$p : \phi$	119.006	<0.001	41.886	<0.001	19.538	<0.001
$p : \eta$	0.779	0.717	0.652	0.991	0.947	0.703
$p : \rho$	1.417	0.116	1.393	0.017	1.234	0.020
$\phi : \eta$	1.026	0.407	1.017	0.443	1.264	0.013
$\phi : \rho$	0.476	0.943	0.570	1.000	0.915	0.801
$\eta : \rho$	0.430	0.972	0.544	0.999	0.797	0.974

Table S 1. Ridge-regression vs lasso. Permutation tests for equality of the group distributions using distance components analysis (lines 3 to 7), and permutation F-tests for the presence of 2-by-2 interactions (lines 8 to 17), using 5, 10, and 15 bins. Results based on 999 permutations. Observe that for all the three different choices of numbers of bins, the results were qualitatively equivalent in the sense that all highly significant results (p-value < 0.001) were preserved across the three number of bins choices.

par.	5 bins obs. stat.	5 bins p-value	10 bins obs. stat.	10 bins p-value	15 bins obs. stat.	15 bins p-value
n	88.776	<0.001	42.231	<0.001	27.331	<0.001
p	107.473	<0.001	61.468	<0.001	44.144	<0.001
ϕ	113.171	<0.001	82.652	<0.001	63.100	<0.001
η	1.313	0.149	1.515	0.023	1.375	0.030
ρ	11.124	<0.001	6.099	<0.001	4.055	<0.001
$n : p$	6.463	<0.001	2.335	<0.001	1.499	<0.001
$n : \phi$	16.226	<0.001	6.994	<0.001	3.952	<0.001
$n : \eta$	0.608	0.880	1.049	0.365	0.959	0.631
$n : \rho$	0.313	0.999	0.675	0.996	0.822	0.972
$p : \phi$	75.628	<0.001	30.782	<0.001	14.471	<0.001
$p : \eta$	1.285	0.187	1.239	0.075	1.027	0.394
$p : \rho$	2.003	0.015	1.685	<0.001	1.483	<0.001
$\phi : \eta$	1.759	0.030	1.417	0.009	1.459	<0.001
$\phi : \rho$	0.529	0.926	0.539	1.000	0.712	1.000
$\eta : \rho$	0.661	0.857	0.739	0.967	0.973	0.587

Table S 2. Ridge-regression vs elastic-net. Permutation tests for equality of the group distributions using distance components analysis (lines 3 to 7), and permutation F-tests for the presence of 2-by-2 interactions (lines 8 to 17), using 5, 10, and 15 bins. Results based on 999 permutations. Except for the $p : \rho$ and $\phi : \eta$ interactions, the results were qualitatively equivalent across the three number of bins choices, in the sense that all highly significant results (p-value < 0.001) were preserved across the three choices.

	5 bins		10 bins		15 bins	
par.	obs. stat.	p-value	obs. stat.	p-value	obs. stat.	p-value
n	167.430	<0.001	78.683	<0.001	51.107	<0.001
p	258.926	<0.001	123.014	<0.001	83.617	<0.001
ϕ	47.080	<0.001	26.688	<0.001	19.560	<0.001
η	0.623	0.837	0.804	0.781	0.790	0.848
ρ	245.826	<0.001	115.291	<0.001	75.513	<0.001
$n : p$	16.689	<0.001	6.341	<0.001	3.314	<0.001
$n : \phi$	6.003	<0.001	2.212	<0.001	1.485	<0.001
$n : \eta$	0.837	0.642	0.771	0.932	0.845	0.941
$n : \rho$	5.548	<0.001	1.821	<0.001	1.353	<0.001
$p : \phi$	37.705	<0.001	9.788	<0.001	4.654	<0.001
$p : \eta$	0.681	0.816	0.652	0.991	0.770	0.996
$p : \rho$	14.939	<0.001	6.168	<0.001	3.597	<0.001
$\phi : \eta$	0.668	0.822	0.735	0.971	0.920	0.797
$\phi : \rho$	1.808	0.029	1.233	0.084	1.187	0.033
$\eta : \rho$	0.443	0.968	0.544	0.999	0.849	0.949

Table S 3. Lasso vs elastic-net. Permutation tests for equality of the group distributions using distance components analysis (lines 3 to 7), and permutation F-tests for the presence of 2-by-2 interactions (lines 8 to 17), using 5, 10, and 15 bins. Results based on 999 permutations. Observe that for all the three different choices of numbers of bins, the results were qualitatively equivalent in the sense that all highly significant results (p-value < 0.001) were preserved across the three number of bins choices.
